# No place like home? A test of the natal habitat-biased dispersal hypothesis in Scandinavian wolves

**DOI:** 10.1098/rsos.181379

**Published:** 2018-12-12

**Authors:** Ana Sanz-Pérez, Andrés Ordiz, Håkan Sand, Jon E. Swenson, Petter Wabakken, Camilla Wikenros, Barbara Zimmermann, Mikael Åkesson, Cyril Milleret

**Affiliations:** 1 Faculty of Applied Ecology and Agricultural Sciences, Inland Norway University of Applied Sciences, Evenstad, 2480 Koppang, Norway; 2 Faculty of Environmental Sciences and Natural Resource Management, Norwegian University of Life Sciences, Postbox 5003, 1432 Ås, Norway; 3 Grimsö Wildlife Research Station, Department of Ecology, Swedish University of Agricultural Sciences, 730 91 Riddarhyttan, Sweden; 4 Norwegian Institute for Nature Research, 7485 Trondheim, Norway

**Keywords:** *Canis lupus*, natal habitat-biased dispersal, habitat availability, habitat selection, individual experience, Scandinavia

## Abstract

Natal dispersal is an important mechanism for the viability of populations. The influence of local conditions or experience gained in the natal habitat could improve fitness if dispersing individuals settle in an area with similar habitat characteristics. This process, defined as ‘natal habitat-biased dispersal’ (NHBD), has been used to explain distribution patterns in large carnivores, but actual studies evaluating it are rare. We tested whether grey wolf *Canis lupus* territory establishment was influenced by the habitat characteristics of the natal territory using the long-term monitoring of the Scandinavian wolf population. We paired the locations of natal and established territories, accounted for available habitats along the dispersing route, and compared their habitat characteristics for 271 wolves during 1998–2012. Wolves with the shortest dispersal distances established in natal-like habitat types more than expected by chance, whereas wolves that dispersed longer distances did not show NHBD. The pattern was consistent for male and female wolves, with females showing more NHBD than males. Chances to detect NHBD increased with the size of habitat defined as available. This highlights the importance of considering the biological characteristics of the studied species when defining habitat availability. Our methodological approach can prove useful to inform conservation and management to identify habitats to be selected by reintroduced or naturally expanding populations.

## 1. Introduction

Natal dispersal influences population dynamics, spatial distribution, genetic structure and the social organization of individuals [1], and plays an important role in the viability of natural populations [2]. Natal dispersal, defined as the movement from the natal area to the site of first potential breeding, most often occurs in the sub-adult stage in birds and mammals [3,4]. During this process, intraspecific competition, mate choice and habitat quality are determinants for each individual's settlement decision [5]. Thus, dispersers may rely on both intrinsic (demographic) and extrinsic (environmental) cues [6,7] to target breeding sites where fitness could be maximized [4,8].

Cues from early life stages obtained in the natal area may help dispersers to quickly estimate the habitat quality and suitability of future settlement locations [9,10]. Local conditions and experience gained during the natal phase may promote phenotypes adjusted to the natal habitat, which may improve fitness if dispersers settle in the same type of habitat [11]. In Siberian flying squirrels *Pteromys volans*, for instance, the use of dreys (twig nests) for nesting after dispersal, instead of using tree cavities, mirrored drey use in the natal site [9]. The advantage would be that dreys help avoiding parasitism, a major reason for changing nests in mammals [9]. Dispersing and eventually settling into a similar habitat is a process termed ‘natal habitat-biased dispersal’ (hereafter, NHBD; [4,9,12,13]). NHBD can thus be defined as a mechanism of habitat selection during the dispersal process that is influenced by the characteristics of the natal territory. NHBD, also termed ‘natal habitat preference induction’ and ‘habitat imprinting’, has been documented for relatively few species (e.g. [10,14]), which promoted calls to study NHBD in more taxa (e.g. [10]). Studies on NHBD in mammals are particularly scarce and have been mostly focused on rodents (e.g. [15]).

Regarding large carnivores, a link has been established between the spatial distribution patterns and the spatial genetic structure of the Canada lynx *Lynx canadensis* [16]. Interestingly, NHBD has been used to explain genetic population differences across landscapes ([13,17], for coyotes *Canis latrans*, [18,19], for different *Canis lupus* populations, [20], for jaguars *Panthera onca*). However, the actual existence of NHBD in large carnivores has been tested only in red wolves *Canis rufus*, which showed NHBD [21], and American black bears *Ursus americanus*, which did not [22]. In red wolves, 71% of pups and 82% of older individuals settled in areas similar to their natal habitats, which may reflect selection for areas with higher prey availability [21]. The social structure, high mobility and territorial behaviour of grey wolves make them good candidates for the study of dispersal [1,23]. However, long-range movements by wolves have rarely been studied at fine scales because of logistical and methodological limitations [24], and because it involves complex relations with habitat characteristics [1,23]. Wolves are generalists in terms of habitat requirements [25], but preferences for flat and forested areas [26], shrub lands [27], and habitats selected by their main prey species [28], for instance, have been documented. Interspecific competition, e.g. with brown bears *Ursus arctos* [29], and general avoidance of human activity [30] can also affect the establishment of wolf territories. Indeed, wolves' avoidance of human activity [30,31] may lead them to select a particular habitat type regardless of the natal habitat characteristics, i.e. several factors may affect whether NHBD exists. However, the long association of young wolves with the parents may improve the ability of young wolves to capture and handle prey in the natal territory [32], which may favour selection of similar habitat types when wolves disperse. Furthermore, NHBD may favour selecting habitats particularly rich in prey (*sensu* [21]).

An essential step to study habitat selection is the definition of habitat availability [33]. For instance, shortage of the natal-like habitat type in the area where an animal disperses to and eventually establishes may limit the occurrence of NHBD. Accounting for availability allows differentiating if the lack of NHBD reflects an individual choice and/or the lack of available habitats. Furthermore, the extent of the area considered available for a dispersing individual may lead to different interpretations on the occurrence of, or lack of, NHBD. Although considering habitat availability is central in habitat selection studies [33], it has rarely been considered when studying NHBD.

The Scandinavian wolf population re-established naturally in the early 1980s and has increased in numbers since 1991 [34]. However, its expansion has been shaped by constraints, such as legal control, poaching [35,36], and low survival of immigrants crossing the Fennoscandian reindeer *Rangifer tarandus* management area, which restricts Scandinavian wolf genetic exchange with the founder population in Finland-Russia. Taking advantage of the detailed, long-term monitoring of the Scandinavian wolf population, we tested the NHBD hypothesis, i.e. we examined if dispersing wolves were influenced by their natal habitat characteristics when choosing a new territory to settle within the distribution range during 1998–2012 in central Scandinavia (electronic supplementary material, figure A1, appendix 1). Using the reconstructed pedigree of this wolf population [34,37], we defined the natal territory as the spatial location of the territory where individuals were born, and the established territory as the location of the territory of the first detected successful pairing with a mate. We studied the similarities between natal, available and established territories, while controlling for habitat availability, by characterizing their landscape attributes with several environmental variables. We predicted that, if Scandinavian wolves perform NHBD, they would be more likely to establish in a territory with habitat characteristics similar to those of the natal territory. Because dispersal distance may play an important role in habitat similarity between natal and established territories [12] and gender influences the dispersal process in many species, with mammals often exhibiting male-biased dispersal [38], we checked if dispersal distance and gender could be important factors to detect NHBD in wolves. Because we did not have access to the actual dispersal trajectories of wolves, i.e. only the location of the natal and established territories was known, we defined habitat availability with different methods, as the definition of availability could also influence the chance of detecting NHBD.

Besides improving our current understanding of wolves' dispersal patterns during a recolonization phase, and providing a new study on the NHBD hypothesis for large mammals, our approach may prove useful for conservation and management in Scandinavia and elsewhere. In particular, information about large carnivores' dispersal patterns is especially important now that several species are recolonizing former ranges in human-dominated landscapes (e.g. [39]).

## 2. Material and methods

### 2.1. Study area

The study area was located in the wolf breeding range in south-central Scandinavia, covering approximately 100 000 km^2^ (electronic supplementary material, figure A1, appendix 1). This area is dominated by boreal coniferous forest mixed with bogs and lakes. The main tree species were Norway spruce *Picea abies* and Scots pine *Pinus sylvestris*, intermixed with birches *Betula pendula* and *B. pubescens* [40]. Secondary land cover types were mires, agricultural lands and human settlements. Human density within the wolf range was low, with less than 1 inhabitant km^−2^ in large areas [41]. Main (paved) road density was 0.19 ± 0.02 km km^−2^, and due to intensive forest management practices, gravel road density was on average 4.6 times higher [42].

The staple prey species for most Scandinavian wolf packs is moose *Alces alces* (e.g. [40,43]) and to a minor extent, roe deer *Capreolus capreolus* [44]. Sympatric large carnivore species in different parts of the wolf range are brown bear [29], Eurasian lynx *Lynx lynx* [45], and wolverine *Gulo gulo* [41].

### 2.2. Data collection

We used data from the long-term wolf monitoring programme in Scandinavia, which is based on a combination of snow tracking, DNA identification and radio-telemetry [34,35,41] to obtain information about successful territory establishment (see electronic supplementary material, appendix 1 for details). We used data from 153 wolf pairs obtained from 1998 to 2012 [36], from annual genetic identification of new Swedish and Norwegian reproductive pairs [35]. During this period, the wolf population increased steadily and numbered 251 individuals (95% CI = 216–299) in the winter 2011/2012 [46].

### 2.3. Definition of successful dispersal

Wolves are territorial and live in packs composed of a breeding, scent-marking pair and their non-marking offspring [32,41]. Most of the offspring disperse from the natal territory, i.e. from the natal pack, either at approximately 1 (70%), or 2 (30%) years old [46]. We defined successful natal dispersal as dispersal from the location of the natal territory to the site where a successful pairing was detected, i.e. the established territory (electronic supplementary material, figure A6, appendix 2). Parentage analysis was based on the microsatellite genotypes identified using scats and urine found during snow-tracking [34]. The pedigree was used to identify the parents of each successful disperser, and therefore identify the location of the natal territory. From the wolf pairs detected during the study period, we identified 271 successful dispersers (140 males and 131 females). For each of them, we defined the spatial location of the parental pair as the centre of the natal territory and the location of the first detected successful pairing as the centre of the established territory.

To define the spatial location of natal and established territories, we used all locations obtained from winter snow tracking of identified individuals in combination with GPS/VHF locations from collared individuals, when available. Because we could not always determine the exact birth year of the successful disperser, we used the centroid of all available locations of the parental pair as the centre of the natal territory and the centroid location of the first detection of successful pairing as the centre of the established territory. We then applied a 1000 km^2^ buffer around each territory centre (i.e. the average wolf home range size; [47]) to approximate the area occupied by the territory [29,36]. For newly established territories detected during the monitoring season (October–February), we assumed that dispersal had occurred six months previous to the detection of the territory, i.e. between the winter monitoring period when the individual was first detected in a pair and the previous monitoring period.

### 2.4. Definition of habitat availability

Because we used the pedigree to obtain the spatial location of the natal and established territory, we did not have access to the dispersal trajectory and the different available habitats encountered by each individual while dispersing. The definition of habitat availability is crucial in habitat selection studies [33]. We therefore tested the influence of different definitions of habitat availability when studying the NHBD hypothesis.

#### 2.4.1. Correlated random walks

**Figure 1.**
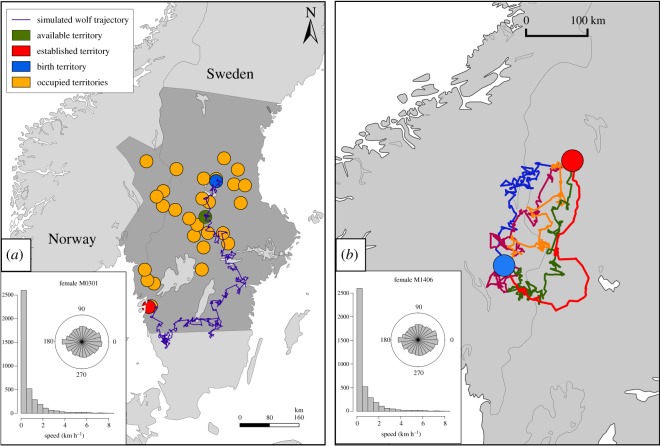
Examples of Correlated Random Walks (CRW) simulations: (*a*) Trajectory (blue line) simulated from the dispersal movement characteristics of the GPS-collared wolf M 03-01 (Lower left corner, from electronic supplementary material, figure A2, appendix 2). The simulated trajectory goes from the natal (blue) to the established (red) territory of a male (G 53-10) that dispersed over a long distance. The locations of the territories occupied 1 year before establishment (*t* − 1) are shown in orange. The randomly selected territory along the simulated dispersal trajectory is shown in green. The study area is shown in dark grey. (*b*) Real dispersing trajectory (red line), from the natal (blue) to the established (red) territory of the GPS-collared wolf M 14-06. The blue, orange, pink and green lines represent examples of CRW simulated by using the dispersing characteristics of the GPS trajectory of the same individual (Lower left corner, from electronic supplementary material, figure A2, appendix 2).

Although the entire study area is in theory available for wolves to establish their territory, wolves are likely to choose among the different habitats encountered during their actual dispersal routes. Therefore, we used available dispersal information from some individuals to create correlated random walks (CRW, [48]) from the natal to the established territories for each of the 271 successful dispersers. CRW allowed us to define habitat availability from an individual perspective. CRW were simulated using movement characteristics of 13 successfully dispersing GPS-collared (Simplex and Tellus collars of Televilt/Followit AB Sweden and GPS-Plus collars of Vectronic GmbH, Germany) wolves in Scandinavia (figure 1; see electronic supplementary material, appendix 2.1 and figures A2 and A3, for detailed information on movement characteristics), using the null model ‘NMs.randomCRW’ from the R package adehabitatLT [49]. Only the GPS locations during dispersal (see electronic supplementary material, appendix 2.1; [46]) were used as observed data to simulate the dispersal trajectory. We used the natal territory of each of the 271 successful dispersers as a starting point, and the established territory as the ending point of the trajectory (see electronic supplementary material, appendix 2.1 for detailed information of the CRW creation). The movement characteristics of each of the 13 wolves during dispersal were used separately to simulate different trajectories and to take into account individual variation in dispersal behaviour [48].

We sampled one random point for each CRW, constraining the creation of the points to the area that could reasonably be available for a disperser, taking into account the breeding range of the species in Scandinavia and the annual location of existing wolf territories (see electronic supplementary material, appendix 2.2). For each successful disperser, we obtained 11 random points extracted from 11 different CRW (electronic supplementary material, appendix 2), which provided a robust sample of matched availability (e.g. [29,42]).

#### 2.4.2. Buffers

CRW have the advantage to use known movement characteristics of wolves during their dispersal. Because it is still a very coarse definition of the habitat available encountered during dispersal, we checked the sensitivity of our results to another definition of availability. We created 11 random points, as the location of potentially available territories, uniformly distributed within buffers created around a straight line drawn between the natal and established territory of each dispersing individual. We sequentially repeated this approach using buffer radius ranging from 25 000 to 300 000 m. We used different buffer sizes around the straight line between natal and established territories to test the sensitivity of our conclusions to the definition of availability (ranging from scenarios where availability was limited around the straight line between the natal and established territory, to scenarios where the whole study area extent was defined as available).

### 2.5. Habitat-related variables

To characterize the habitat characteristics of natal, available and established territories, we applied a 1000 km^2^ circular buffer around each territory centre [29,36], and extracted all habitat variables described below (table 1).

**Table 1.** Summary of habitat variables used to characterize the Scandinavian grey wolf territories and respective sources of information. GIS layers were converted to 1 km × 1 km grid cells.

landscape variables	description	source
interspecific		
bear density	kernel density estimator based on records of shot bears	[29]
moose density	annual harvest density at municipality/management unit	www.viltadata.se, Sweden; www.ssb.no, Norway
human		
human density	no. of inhabitants per km^2^	www.scb.se, Sweden; www.ssb.no, Norway
main road density	km of main roads per km^2^	1:100 000 Lantmäteriet, Sweden; N50 kartdata, Staten-skartverk, Norway
secondary road density	km of gravel roads per km^2^	1:100 000 Lantmäteriet, Sweden; N50 kartdata, Staten-skartverk, Norway
remoteness and accessibility	combination of building and road densities per km^2^	[29,46] 200 m × 200 m
land cover		
vegetation	percentage of forest, mires, mountains, human-dominated areas, water and agricultural areas.	[47]; Swedish Corine land cover map Lantmäteriet, Sweden, 25 m × 25 m merged with Northern Research Institute's vegetation map, Norway, 30 m × 30 m into a 25 m × 25 m raster.
altitude	altitude in metres above sea level	DEM 25 m × 25 m; Geographical Data Sweden, Lantmäteriet; Norge digital, Statens kartverk, Norway
slope	slope in degrees	
roughness	difference in m between the maximum and the minimum value of a cell and its 8 surrounding cells	

*Prey density:* moose harvest is a robust, but delayed indicator of moose density, because temporal variation in harvest density is better explained by moose density in year *t* − 1 than in year *t* [50]. Therefore, we used moose harvest density (number of moose harvested/km^2^) at the municipality and management unit level in Scandinavia with a 1-year time lag [29,36].

*Brown bear density:* evidence of competition between wolves and bears has been shown in Scandinavia [29,51,52]. We used an index of bear density ranging between 0 and 1, denoting low and high density respectively, based on records of shot bears [29].

*Human-related variables:* we obtained human density (inhabitants km^−2^) at the municipality level. We also used density of main (paved) and gravel roads (km km^−2^) and an index of human accessibility of the landscape, based on combined building and road densities (number of buildings along km of road stretches) [29,36].

*Land-cover variables*: we used a vegetation map (table 1) that included the most important habitat types that are known to affect wolf habitat selection in Scandinavia [29]: forest, mire, mountain, water, agricultural areas and human-dominated areas. We used a 3 × 3 moving window with a resolution of 200 m to calculate the percentage of each vegetation class. We merged the Digital Elevation Model (DEM) of Sweden and Norway. We also computed the slope (degrees) and roughness at a 25-m resolution by using the ‘terrain’ function (R package raster; [53]) from the DEM layer and a 5 × 5 moving window.

*Wolf density*: We used the density of wolf pairs within a 40 km radius buffer [29,36,47] as a proxy for wolf density.

### 2.6. Definition of habitat similarity

One of the keystones of our study was identifying habitat similarity among natal, established and available territories. First, we performed a Principal Component Analysis (PCA) on the matrix containing the environmental variables characterizing the natal, available and established territories. We also included year as a continuous variable to control for a potential time effect. All variables were standardized. Second, we used the *K*-means clustering method over the five Principal Components of the PCA to group wolf territories with similar habitat characteristics in 6 clusters (electronic supplementary material, tables A1 and A2; appendix 3; figure A10, appendix 5). Therefore, each cluster contained natal, available and established territories that shared habitat similarities.

To ensure that our habitat similarity definition did not influence our ability to detect NHBD, we tested whether alternative clustering methods and a variable number of clusters would affect our conclusions, by using *K*-means over a number of clusters ranging from 4 to 10. In addition, we also used Partition Around Medoids (PAM), hierarchical clustering methods, and a distance metric of similarity to the natal habitat (see electronic supplementary material, appendix 4) to define habitat similarity.

### 2.7. Statistical analyses

We used conditional logistic regression with the binary response established (1) and available (0) for each individual to test whether or not individuals established in territories with habitat characteristics similar to their natal territories (R package survival; [54]). The established territory was paired with the 11 available territories (1 : 11) and each individual was used as a ‘stratum’ [55]. We created the NHBD binary variable denoting whether each available and established territory of a focal individual were assigned to the same cluster as the natal territory. A positive beta estimate for the variable NHBD would confirm the NHBD hypothesis. Because of the potential influence of dispersing distance (electronic supplementary material, figure A5, appendix 2) on habitat selection (e.g. [56]), we tested the NHBD hypothesis for short (less than 40 km), medium (40–200 km) and long (more than 200 km) dispersing wolves. We also tested the influence of sex in NHBD. We chose to discretize the dispersing distance variable to avoid having to compute a three-way interaction between the NHBD*sex*distance, while controlling for nonlinear effects of dispersal distance. We included the habitat variables described above to control for avoidance/selection of specific habitat types. We tested for correlation among variables and excluded human-dominated areas, agricultural lands, altitude, roughness, mires and secondary roads from all models (Pearson coefficient > 0.6). Additionally, we tested whether wolf density could affect NHBD by including an interaction term between wolf density and the variable NHBD. We checked for NHBD using conditional logistic regression over a different number of clusters, different clustering methods, and a metric of similarity to the natal territory (electronic supplementary material, appendix 4), and under different definitions of habitat availability. We checked whether individuals significantly selected or avoided the natal habitat type by using *p*-values < 0.05 as a threshold. All analyses were conducted in R v. 3.1.1 [57].

## 3. Results

### 3.1. NHBD

**Figure 2.**
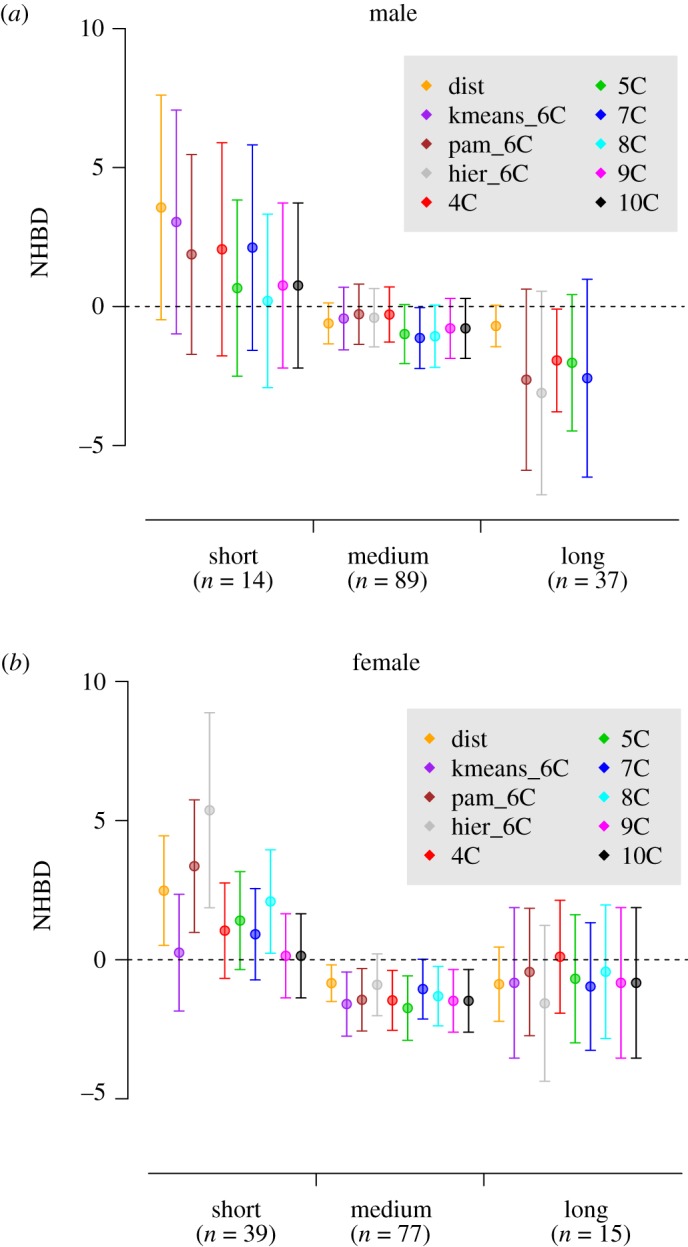
Coefficients (*β*) and 95% confidence intervals (CI) of NHBD for male (*a*) and female (*b*) grey wolves in Scandinavia (1998–2012). Values > 0 represents selection and values < 0 represents avoidance of habitat characteristics similar to the natal one when establishing. Availability was quantified by using CRW. Parameters were estimated from the conditional logistic regressions for short (less than 40 km), medium (40–200 km) and long (more than 200 km) dispersers. For each dispersing distance category the NHBD *β* coefficients for the different methods of defining habitat similarity are represented by different colours: Distance similarity metric (dist), cluster division from 4 to 10 clusters with (*K*-means kmeans_6C, 4C–10C), and 6-cluster division with Hierarchical Clustering Methods (hier_6C) and PAM (pam_6C). For long dispersers, 8C and 9C are not shown in the Figure, because of convergence issues. See electronic supplementary material, appendix 4 for further details on the use of different habitat similarity metrics. The β values for the dist metric were multiplied by 100 for readability purposes in this figure.

Using the CRW approach to define availability, wolves from all dispersing categories had access to available territories that had both similar and different habitat characteristics compared to their natal territory (electronic supplementary material, figure A4, appendix 2). Wolves that dispersed short distances from their natal territories showed NHBD (figure 2), with short-dispersing females showing the highest NHBD coefficients (figure 2*b*). There was no evidence of NHBD for medium and long dispersers (figure 2), with long-dispersing males showing the strongest selection against their natal habitat (figure 2*a*). These conclusions were consistent, regardless of the method used to define habitat similarity (figure 2).

**Figure 3.**
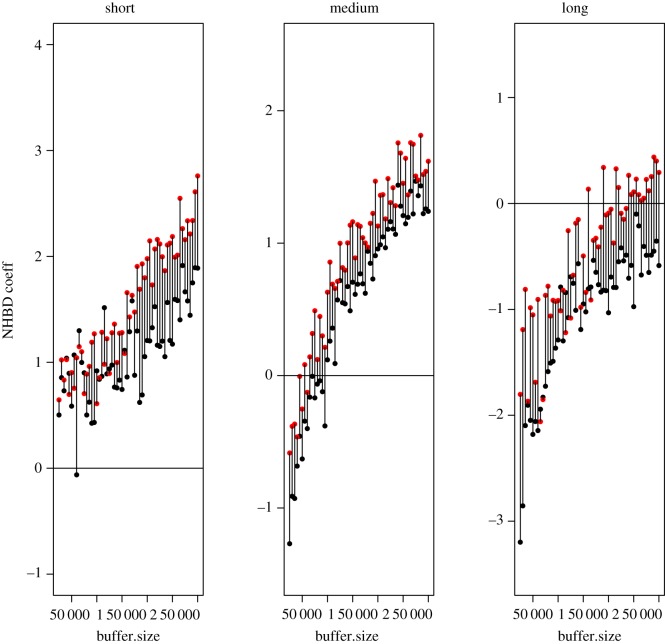
Coefficients (*β*) of NHBD for male (black dots) and female (red dots) grey wolves in Scandinavia (1998–2012). Values > 0 represent selection and values < 0 represent avoidance of habitat characteristics similar to the natal ones when establishing. Vertical segments link female and males coefficients for a specific buffer size for a better visual interpretation of sex differences. Parameters were estimated from the conditional logistic regressions for short, medium and long dispersers. Availability was defined by using different buffer sizes. Habitat similarity was defined by using *K*-means with a 6-cluster division (see electronic supplementary material, figure A9; appendix 4 for results with the different methods of defining habitat similarity).

Using the buffer approach to define habitat availability, the chance of detecting NHBD increased when the available territories were sampled within larger buffer sizes (figure 3). This pattern was consistent for all dispersing distances and for both sexes, with females having consistently higher NHBD coefficients than males (figure 3). Long-distance dispersers did not show NHBD (figure 3), regardless of the buffer size used, confirming the results provided by the CRW approach (figure 2). As with the CRW, conclusions were consistent regardless of the method used to define habitat similarity (electronic supplementary material, figure A9; appendix 4).

### 3.2. Influence of habitat variables on wolf territory establishment

Wolves selected forest (*β* = 9.21; 95% confidence interval (CI) = [5.87, 12.55]; table 2) and mountain areas (*β* = 11.45; CI = [6.70, 16.20]; table 2) when establishing a territory. Wolves avoided high slopes (*β* = −0.70; CI = [−0.96, −0.43]; table 2), areas with higher human accessibility (*β* = −0.01; CI = [−0.01, −0.004)]; table 2) and areas with high bear density (*β* = −1.20; CI = [−2.12, −0.28)]; table 2). Higher wolf density increased the probability of establishment (*β* = 0.36; CI = [0.24, 0.49]; table 2), but it did not show any interactive effect with NHBD (*β* = −0.03; CI = [−0.20, 0.14]; table 2).

**Table 2.** Coefficients (*β*) and 95% confidence intervals (CI) of the probability of establishing a territory for grey wolves in Scandinavia (1998–2012). Habitat availability was defined by using CRW and habitat similarity was defined by using *k*-means with 6 clusters division. Parameters are estimated from the conditional logistic regression of all dispersal distances. Wald test scores (*z*) and *p*-values are included for each variable, with significant variables given in italics.

variables	*β*	95% CI	*Z* score	*p*-value
NHBD	−0.34	[−0.95 to 0.27]	−1.09	0.28
wolf density	*0.36*	[*0.24 to 0.49*]	*5.64*	*<0.001*
human density	0.004	[−0.001 to 0.01]	1.70	0.09
forest	*9.21*	[*5.87 to 12.55*]	*5.40*	*<0.001*
water	1.85	[−1.94 to 5.63]	0.96	0.34
mountains	*11.45*	[*6.70 to 16.20*]	*4.73*	*<0.001*
main road	−0.93	[−3.59 to 1.73]	−0.69	0.49
bear	*−1.20*	[*−2.12 to* *−0.28*]	*−2.55*	*<0.05*
slope	*−0.70*	[*−0.96 to* *−0.43*]	*−5.12*	*<0.001*
human accessibility	*−0.01*	[*−0.01 to* *−0.004*]	*−3.49*	*<0.001*
moose density	−0.87	[−2.44 to 0.71]	−1.08	0.28
NHBD*wolf density	−0.03	[−0.20 to 0.14]	−0.34	0.73
NHBD*sex	*−0.80*	[*−1.41 to* *−0.20*]	*−2.63*	*<0.01*

## 4. Discussion

Detecting NHBD in Scandinavian wolves depended on individual wolf dispersal distances from the natal territory and on the definition of habitat availability. Whereas our results show some support for the NHBD hypothesis, they make it conditional on both biological traits (i.e. wolf dispersal distance and sex) and the methodological approach used for defining habitat availability. Generally, the chances of detecting NHBD increased with the size of the area that was defined as available for a given individual to establish its territory. However, short-dispersing individuals were more prone to select habitat similar to their natal territory, regardless of the methods used (i.e. habitat similarity and habitat availability definitions, figures 2 and 3). This pattern was generally consistent for both male and female wolves, with females showing NHBD more than males (figure 3). Besides novel results in terms of NHBD, Scandinavian wolves selected for forested, mountainous terrain, and areas inhabited by other wolves, but avoided steeper terrain, areas with higher human accessibility, and areas inhabited by bears (table 2) when establishing their territories. These results are in agreement with previous studies on wolf habitat selection [26,28,29].

Short-dispersing Scandinavian wolves established in natal-like habitat types more than expected by chance, whereas wolves that dispersed medium and long distances did not show NHBD (figures 2 and 3; see electronic supplementary material, appendix 2 for definition of dispersal distances). The chance of finding similarity between natal and established territories is linked to dispersal distance, as availability of the natal habitat type is generally higher nearby the natal territory (electronic supplementary material, figure A8, appendix 3; [12,58]). Therefore, regardless of what is causing individual variation in dispersal distances [24,41], a long-dispersing individual is less likely to find available habitat similar to its natal habitat. A long-dispersing individual has likely encountered similar habitat types during the first stage of its dispersal, i.e. close to its territory, as allowed by our definition of availability, but it may simply not be able to perform NHBD, because of a lack of similar habitat available once it has moved far away from its natal territory. On the contrary, an individual having a short dispersal distance is more likely to find habitat available similar to its natal habitat. Therefore, in order to perform NHBD, an individual should likely stay in the vicinity of its natal territory. There might be various reasons explaining inter-individual variation in dispersal distance [38], and we did not specifically test whether NHBD is one of them. However, the fact that short-dispersing individuals tended to favour habitat similar to their natal territory, suggests that NHBD is an arguable reason for short dispersal distance.

Wolves may use cues (experience) obtained in the natal habitat to reduce the time invested in exploring new environments [11], but the time span in which these cues are useful may be limited, and therefore this mechanism might only be effective for wolves dispersing short distances. Therefore, finding NHBD only for short dispersers could reflect both habitat availability and behavioural responses. The ability to handle, capture and process food is improved by experience in the natal habitat [11] and may explain the presence of NHBD. Wolves live in packs and the long association with the parental pair could increase offspring opportunities to learn about components of hunting behaviour that are not innate [32]. This learning process may favour the selection of natal-like habitat and/or prey types, and this was the reason used to explain why red wolves selected habitats rich in prey and similar to their natal areas [21]. Although the wolf is considered a generalist species, prey and habitat specialization have also been shown in a grey wolf population, where two genetic clusters were associated with two different habitat types [19]. Beyond the potential mechanism explaining NHBD for short dispersers and its lack for medium and long dispersers, our results reinforce the importance of considering the effect of dispersal distances when studying habitat selection [56].

Mammals often exhibit male-biased dispersal [38], and in our study there was indeed a larger number of female wolves dispersing short distances (39 females, 14 males), a similar number of males (*n* = 89) and females (*n* = 77) dispersing medium distances, and a higher number of males dispersing long distances (37 males, 15 females; figure 2). Females showed more NHBD than males for all our definitions of habitat availability (figure 3), resembling the pattern found in many mammals, with females often being more philopatric to their natal habitats than males (e.g. [3]).

Quantifying habitat availability is central in habitat selection studies. Previous research has shown bias in habitat selection results when the analytical method does not take the animal's functional response into account, i.e. its choices depending on what is available [59]. In our study, chances to detect NHBD increased with the size of the area defined as available (figure 3), which probably included a larger, more contrasting gradient of habitat types (e.g. see the distribution of clusters of habitat types in electronic supplementary material, figure A8, appendix 3) than smaller areas. An ideal way to deal with the problem of availability definition in studies of habitat selection during dispersal would be to account for habitat availability within the observed dispersing route of each individual (e.g. GPS-based trajectories). Including this information is frequently unfeasible in financial and logistical terms, and particularly challenging for secretive animals [59], so studies integrating dispersal behaviour and availability are valuable (e.g. [60]).

The correlated random walks (CRW) that we used are a simplification of an individual dispersal behaviour, because CRW do not account for habitat selection during the dispersal process. However, we suggest that sampling availability with CRW may reflect how a wolf, in this case, encounters and eventually selects habitat better than arbitrary buffers that lead to varying NHBD results, yet highlighting useful patterns (figure 3). CRW informed by the routes between the natal and the established territory of GPS-collared wolves also avoided the inclusion of habitats that, while being theoretically available for a dispersing wolf, are not likely to be encountered during its dispersal process, which often occurs in straightforward routes (electronic supplementary material, figures A2 and A3, appendix 2).

Nevertheless, we also created progressively larger buffer sizes as an alternative method to quantify habitat availability and its potential influence on NHBD. The combination of wolf dispersal distances and different scenarios of habitat availability led to some variation in the magnitude and direction of the NHBD response in Scandinavian wolves (figures 2 and 3), which reinforces the importance of defining availability [33] and the call for caution when interpreting habitat selection results [59]. Quantifying habitat availability for dispersing individuals is indeed a recognized challenge in habitat selection studies [61]. The few, previous attempts to test NHBD on large carnivores did not quantify habitat availability, but used a proxy of land cover types in natal and established territories [21], or applied a previous map of habitat suitability [22]. Interestingly, our analyses showed that individuals with short dispersal distances performed NHBD more than medium and long dispersers did (figures 2 and 3), regardless of our alternative definitions of habitat availability.

The habitat selection of wolves establishing territories, i.e. selecting for forested, mountainous terrain, and areas inhabited by other wolves, and avoiding steeper terrain, areas with higher human accessibility, and areas inhabited by brown bears (table 2), is consistent with previous studies on wolf habitat selection in Scandinavia [29,31,42] and elsewhere [26,28,62]. Several studies have actually shown the negative influence of humans on wolf occurrence [29,31] and mortality [35,36,41] in Scandinavia and other areas [30]. The high impact that human activities have on Scandinavian wolves, e.g. through poaching, legal control actions, and low survival outside the breeding range [35,36,41], likely masks or overrides cues obtained on their natal habitats, which in turn can reduce the probability of finding a stronger pattern of NHBD in this wolf population. For instance, individuals might select for habitats particularly rich in prey, but such areas could be avoided, rather than selected, if human activity levels are just too high.

NHBD has rarely been reported for wildlife in general [63,64], and has most often been documented in highly heterogeneous habitats ([9,12], but see [64]). To test for NHBD in Scandinavian wolves, we used different habitat classifications through different clustering methods and number of clusters. The observed pattern on NHBD remained consistent with different methods of habitat similarity definition (figure 2; electronic supplementary material, figure A9, appendix 4), which reinforces the robustness of that pattern. We selected the habitat types representing the most important factors that affected wolf habitat selection in Scandinavia [29]. However, since habitat selection is a hierarchical process acting at several spatio-temporal scales [33], there might be other important habitat types that were not captured by our definition of habitats at the spatial scale of our study.

We tried to optimize information on natal and established territories extracted from genetic pedigrees to mirror the dispersal mechanism, accounting for dispersal distances [56], and including prior knowledge of wolf behaviour when defining availability. We encourage other studies to further account for more factors that may affect dispersal, e.g. the landscape context dependency [65], and individual variation in wolf behaviour and physiological state [32,66,67]. We also suggest that performing our methodological approach for carnivore populations inhabiting more heterogeneous landscapes offers potential to reveal if NHBD occurs. In southern Europe, for instance, wolf populations range from high, forested mountains to lower, mostly treeless agricultural areas, therefore exhibiting contrasting diets (e.g. [68,69]). Such habitat heterogeneity may help to understand the role of different landscape features in the dispersal process of wolves and other large carnivores across their distribution range. Indeed, testing for NHBD with spatial locations of natal and established territories extracted from genetic pedigrees and/or using locations of GPS-collared animals can provide essential information for conservation and management. For instance, to delineate sensitive areas and inform reintroduction or translocation programmes about the habitat types that will be more likely occupied by released animals (e.g. [10,11]); and to forecast expansion fronts of currently expanding populations of large carnivores. We therefore encourage researchers to proceed with similar studies, as they can improve our ecological understanding of dispersal processes, while providing essential information for practitioners.

## Supplementary Material

Appendix

## Data accessibility

Data available from the Dryad Digital Repository at: http://dx.doi.org/10.5061/dryad.76hv890 [70].
