# Supplementary material for: No place like home? A test of the natal habitat-biased dispersal hypothesis in Scandinavian wolves
Source: R Soc Open Sci. 2018 Dec 12;5(12):181379. doi: 10.1098/rsos.181379 (PMC6304128; doi:10.1098/rsos.181379)
Supplement: Appendix [file rsos181379supp1.docx]

APPENDIX

No place like home?

**A test of the natal habitat-biased dispersal hypothesis in Scandinavian wolves**

Ana Sanz-Perez^1^, Andrés Ordiz^2^, Håkan Sand^3^, Jon E Swenson^2,4^, Petter Wabakken^1^, Camilla Wikenros^3^, Barbara Zimmermann^1^, Mikael Åkesson^3^, Cyril Milleret^1^

**APPENDIX 1**

**STUDY AREA AND METHODS**

Norwegian and Swedish wildlife management authorities conducted extensive wolf monitoring based on snow tracking every winter, when observations of territorial scent markings and estrous blood were recorded and collected to locate and distinguish among wolf territories (1). DNA analyses allowed the reconstruction of a quasi-complete pedigree of the population, based on invasive samples, including tissues from retrieved dead wolves and blood from captured wolves and non-invasive samples, including primarily hair, scats, and urine (1-3). Additionally, the Scandinavian wolf research project (SKANDULV) captured and fitted VHF and GPS collars on 107 individual wolves during the period 1998 - 2012 (4-6). The Swedish GPS data were deposited into the Wireless Remote Animal Monitoring (7) database system for data validation and management.


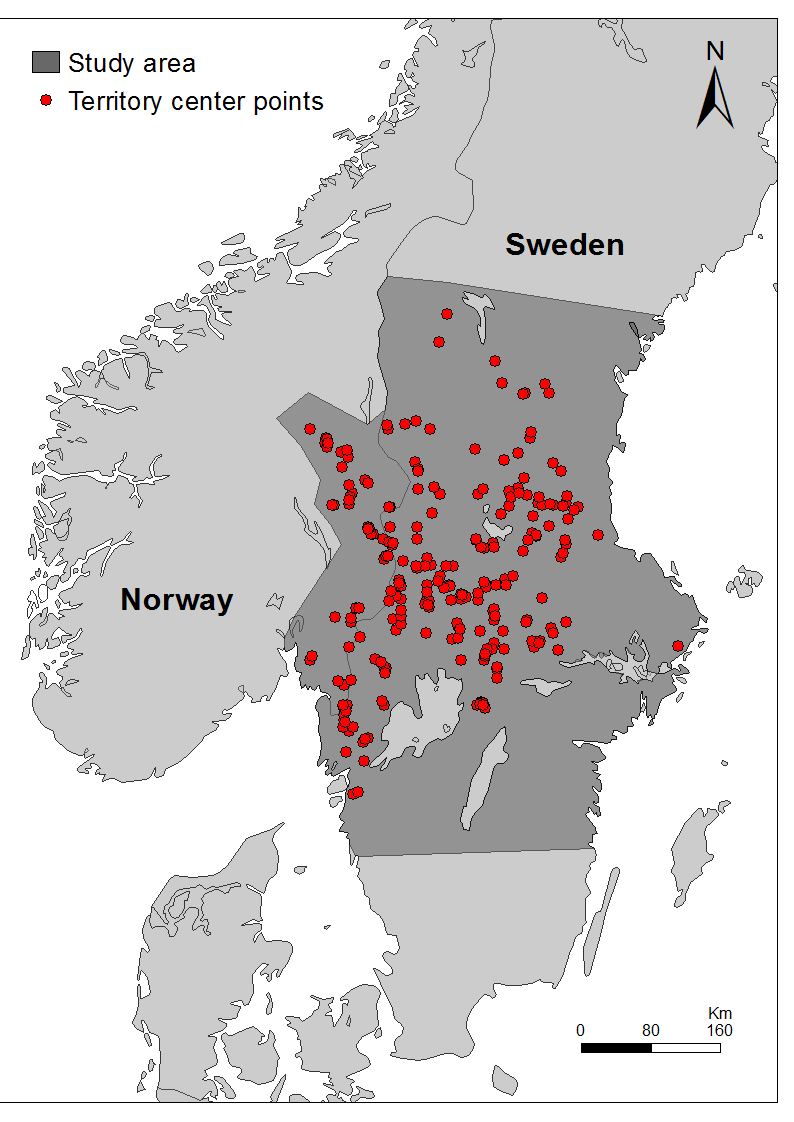


**Figure A1**. Map of the study area in Scandinavia, showing the center points of all wolf natal and established territories detected between 1998 and 2012.

**APPENDIX 2:**

**DEFINITION OF HABITAT AVAILABILITY USING CORRELATED RANDOM WALKS (CRW)**

**2.1. Random walks**

When creating random walks, defining an arrival point is challenging. We therefore simulated random walks until one of them crossed the established territory, i.e., the random walk crossed the 1000 km^2^ buffer created around the center of the established territory. Simulated trajectories from GPS-collared individuals with short dispersal distances had movement characteristics that did not allow the trajectory to reach the established territory for individuals having long dispersal distances. Classified from straight-line distances, we therefore characterized GPS-collared wolves with short-, medium-, and long-dispersing distances to simulate short (< 40 km, 53 individuals), medium (40 - 200 km, 166 individuals) and long (> 200 km, 52 individuals) dispersal trajectories between natal and established territories (see Fig. A5 for distribution of dispersal distances). Short dispersal characterized individuals that established a territory that bordered their natal territory. The threshold of 40 km was selected, because it is slightly greater than the doubled radius of a hypothetical circular wolf territory of 1000 km^2^, i.e., the average wolf home range size in Scandinavia (6). The trajectories from natal to established territories of short dispersing individuals were simulated with the movement characteristics of two of the 13 GPS-collared wolves, i.e., the GPS-collared wolves with shortest dispersal distances (Fig. A3). Preliminary analyses showed that, to simulate the trajectories from natal to established territories of long dispersing individuals (> 200 km), we could only use movement characteristics of the three GPS-collared wolves that performed long natal dispersal, so that the simulated trajectory reached the established territory (Fig. A2). We then used the 11 GPS-collared wolves with the longest and medium dispersal distances (Fig. A2) to simulate the trajectories with medium distances between natal and established territories. From the 13 GPS-collared wolves, we used locations during dispersal from the approximate dispersal date to the settling date for all individuals, except for Female M 03-01, Male M 14-08, and Male M 09-12. For these wolves, we used positions from the dispersal date to the date when the GPS-positioning system stopped functioning.

**2.2. Create random points from CRW**

We sampled one random point for each CRW, constraining the creation of the points to:

1) The actual wolf breeding range in 1998 - 2012, which is limited by management to south-central Scandinavia, to reduce or prevent conflict with reindeer and sheep husbandry (8). Most of the dispersing wolves that roam out of the breeding range are shot before finding a partner and establishing a territory, thus we prevented random points from falling outside the wolf breeding area (Fig. A1, Appendix 1).

2) Availability changed annually, because territory occupancy was dynamic and wolf pairs cannot settle in territories already occupied by another pair. The two possibilities for a wolf to settle within an existing territory is to outcompete the same-sex individual of the existing wolf pair or take a vacant place in a territory, i.e., due to natural or human-caused death. Because intraspecific competition in Scandinavian wolves seems rather low (9), we did not allow random points to be located within territories that were occupied in the year before establishment (*t-1*), which likely represented the situation of the year of establishment (*t*). However, if a turn-over or the death of the individual composing the territory was documented between *t-1* and *t*, the location of the random point within this territory was allowed (10).


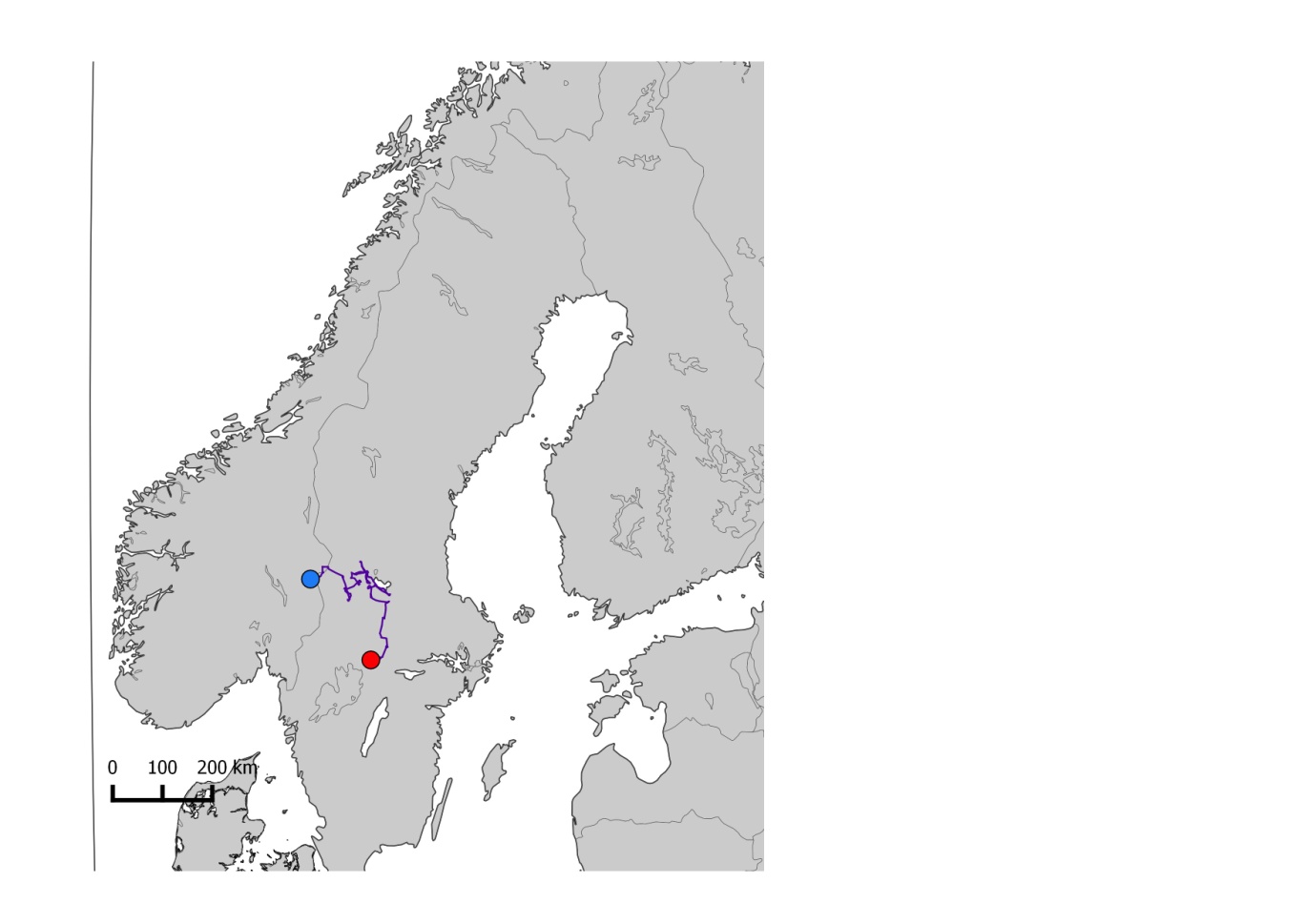

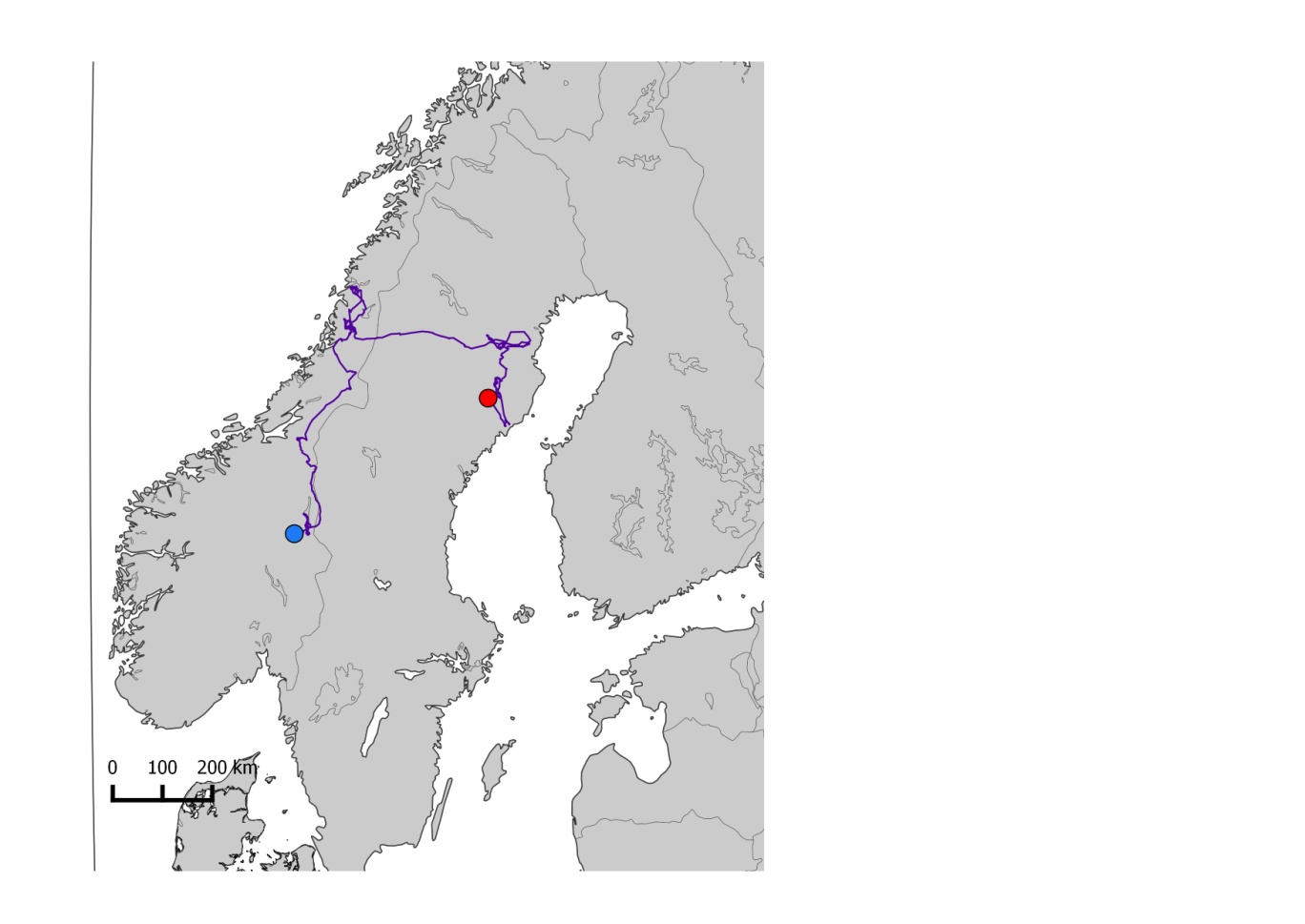

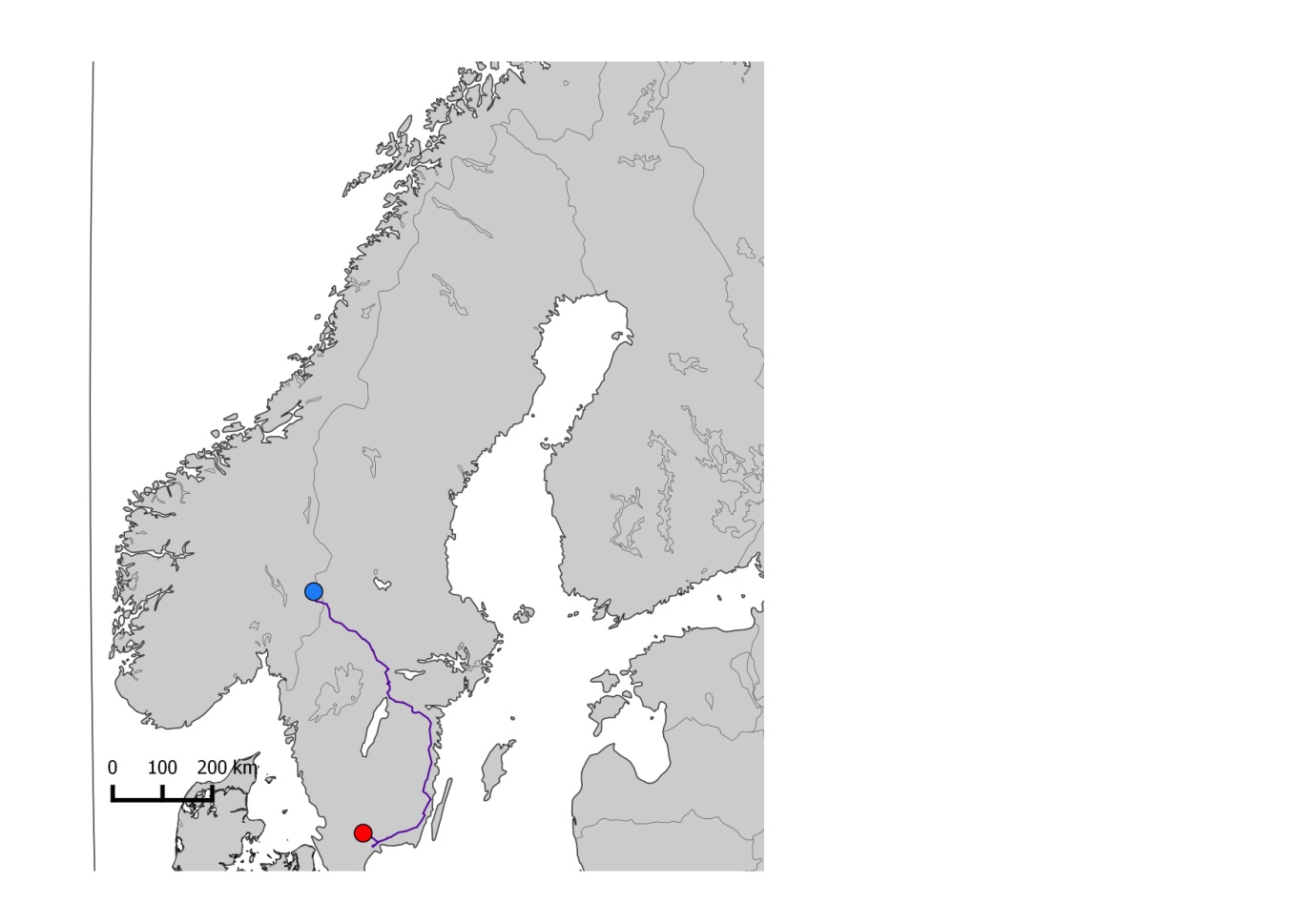


**Male M 11-12**

**Male M 14-08**

**Female M 11-04**

**Female M 03-01**

**Female M1408**


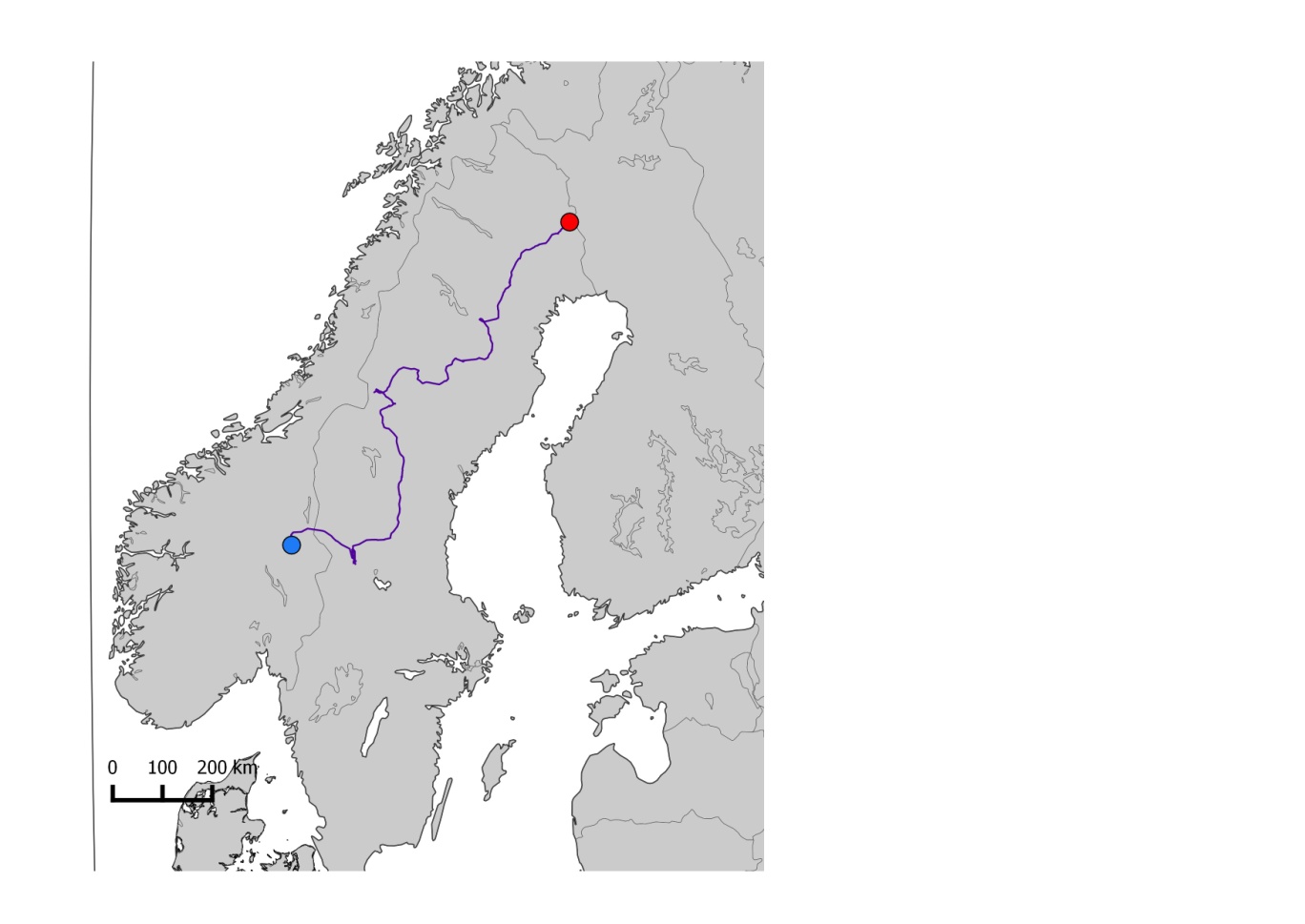

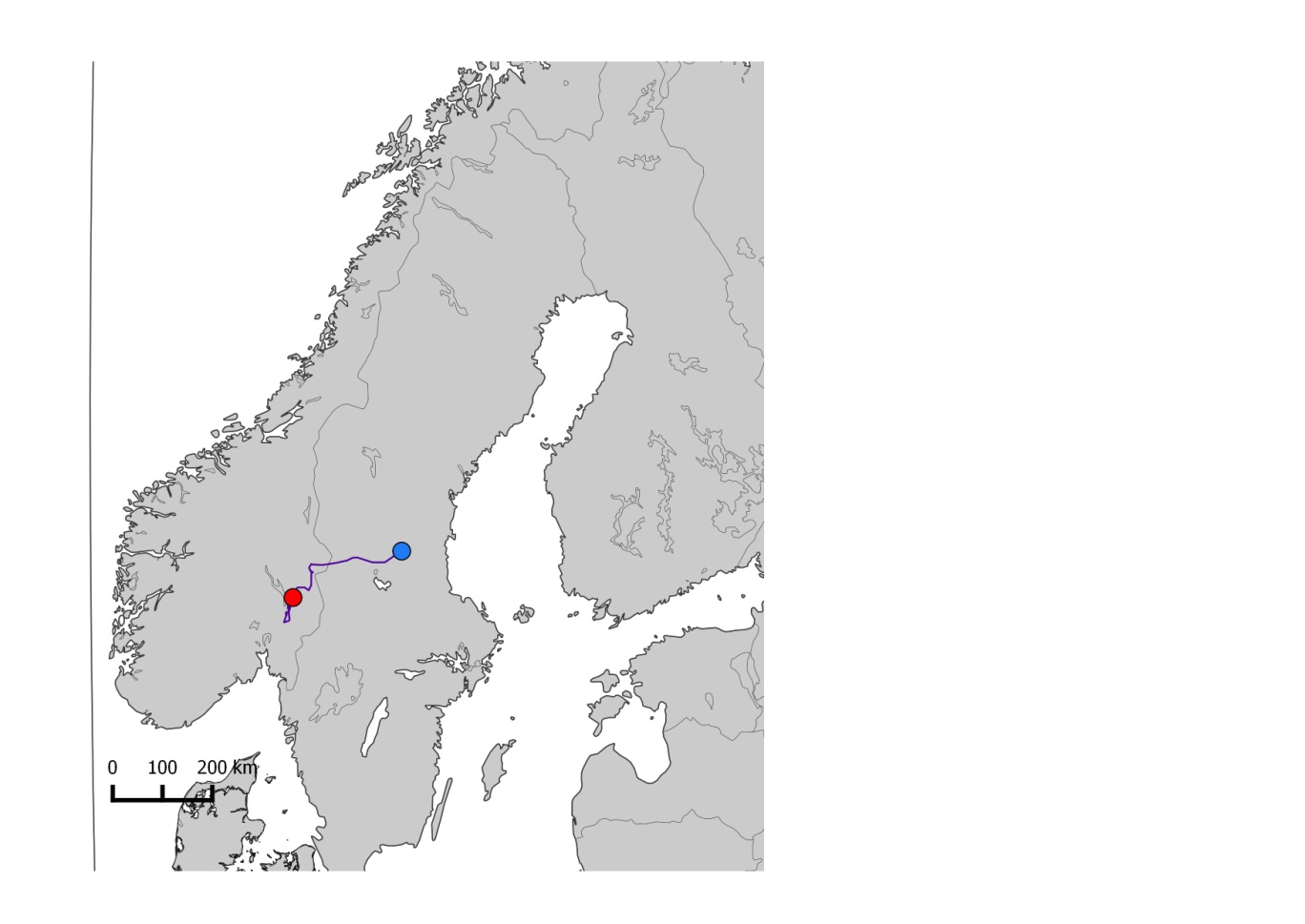

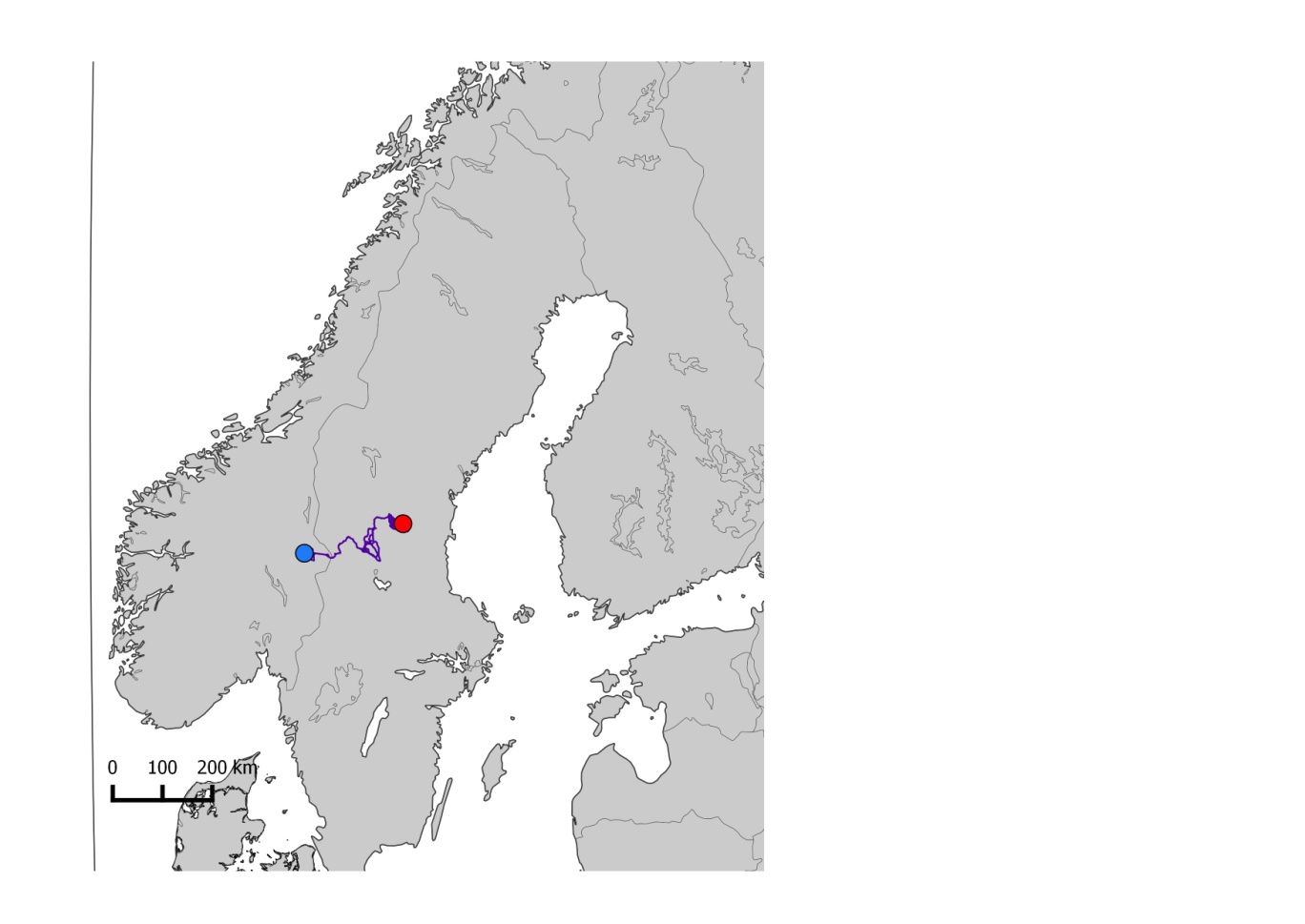

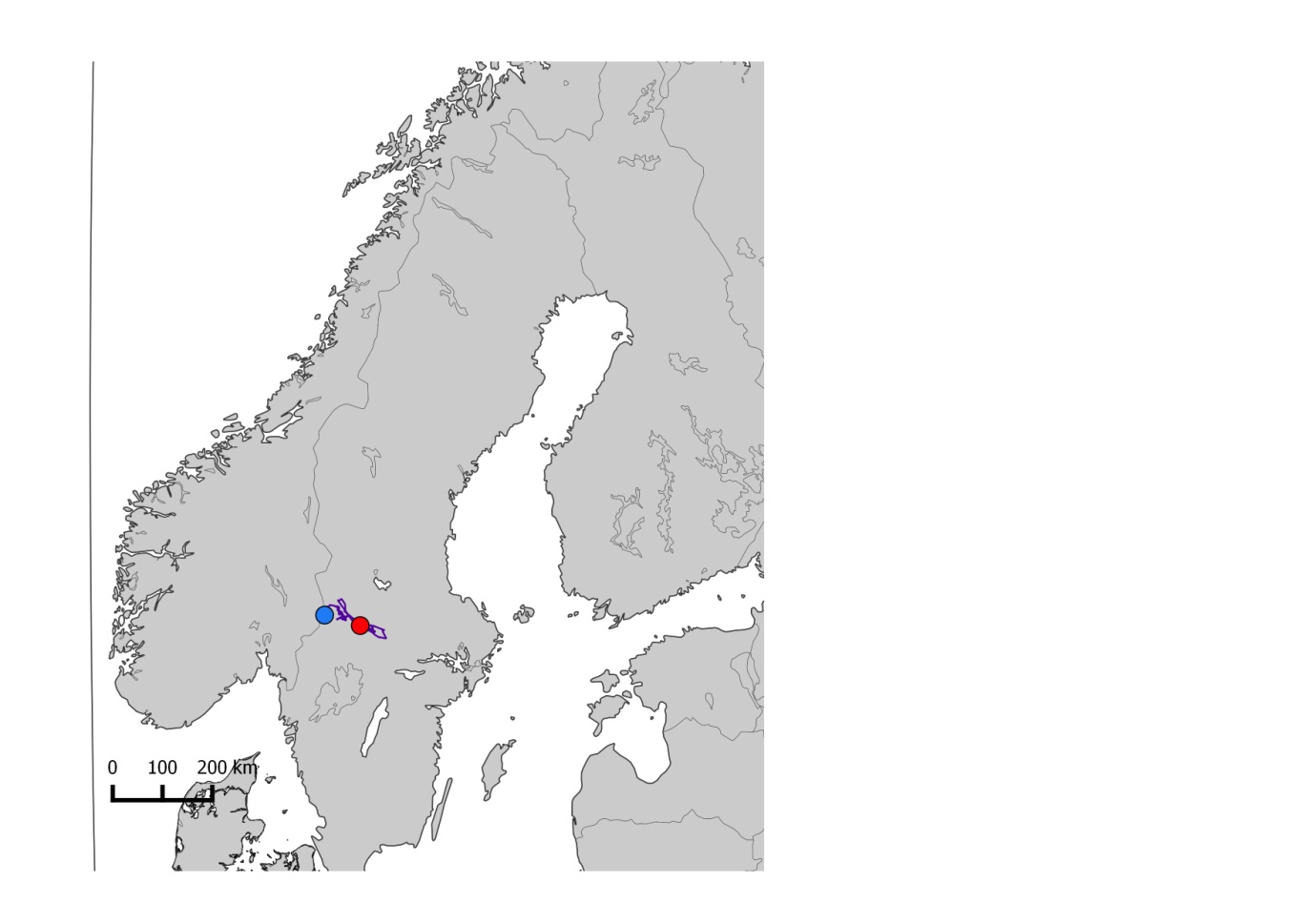

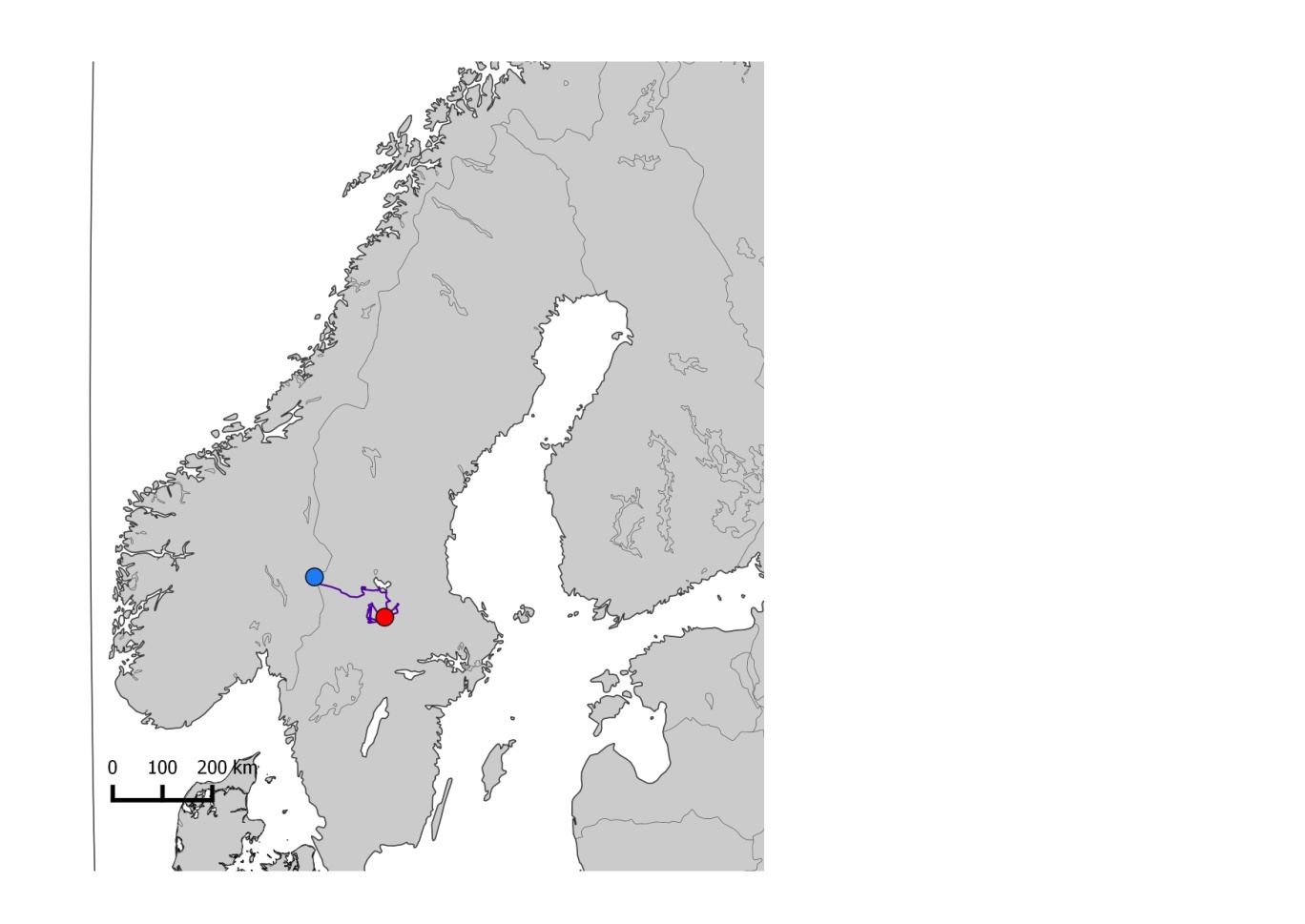
1

**Female M 06-09**

**Female M 09-02**

**Female M 11-05**

**Female M 03-02**

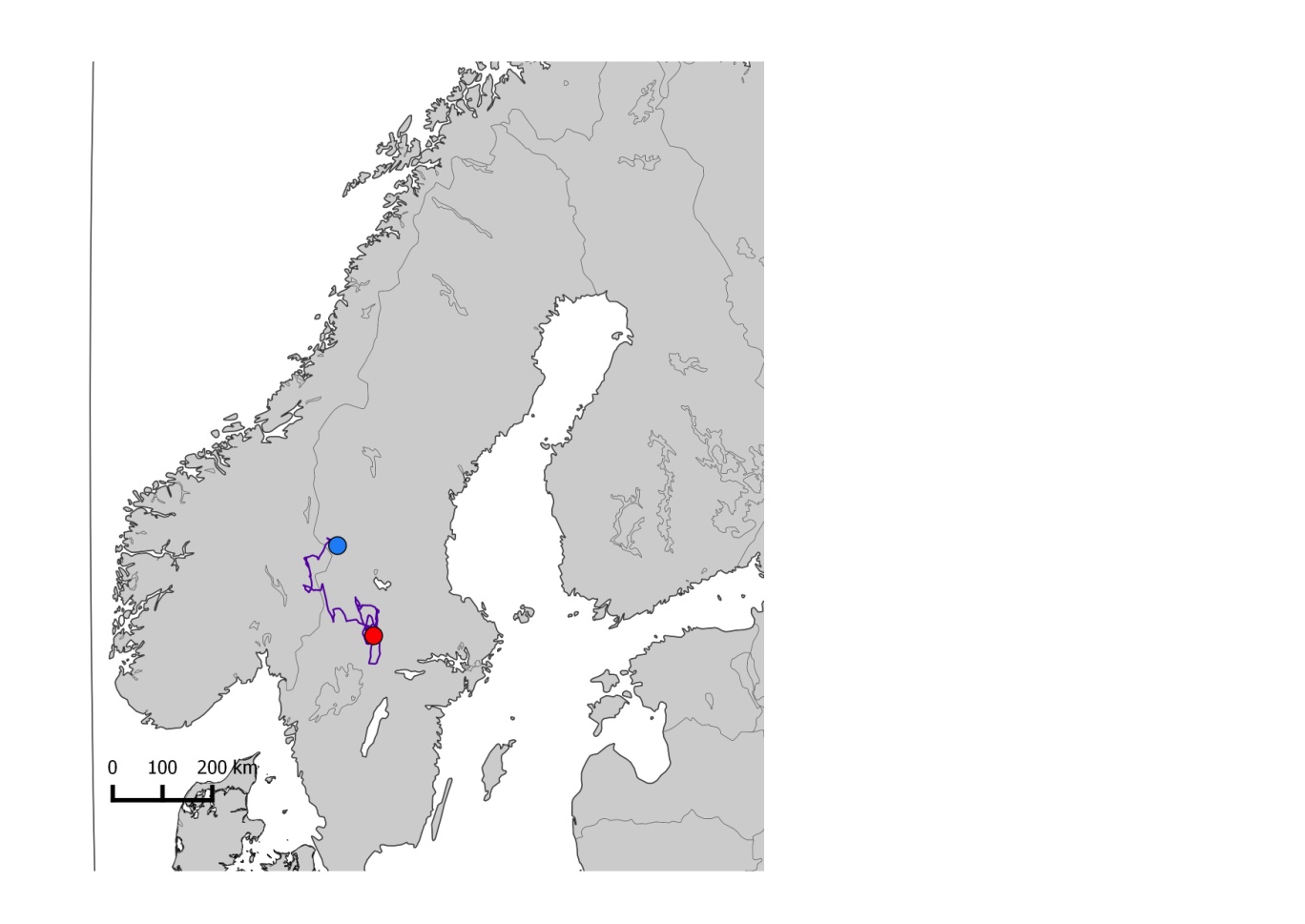

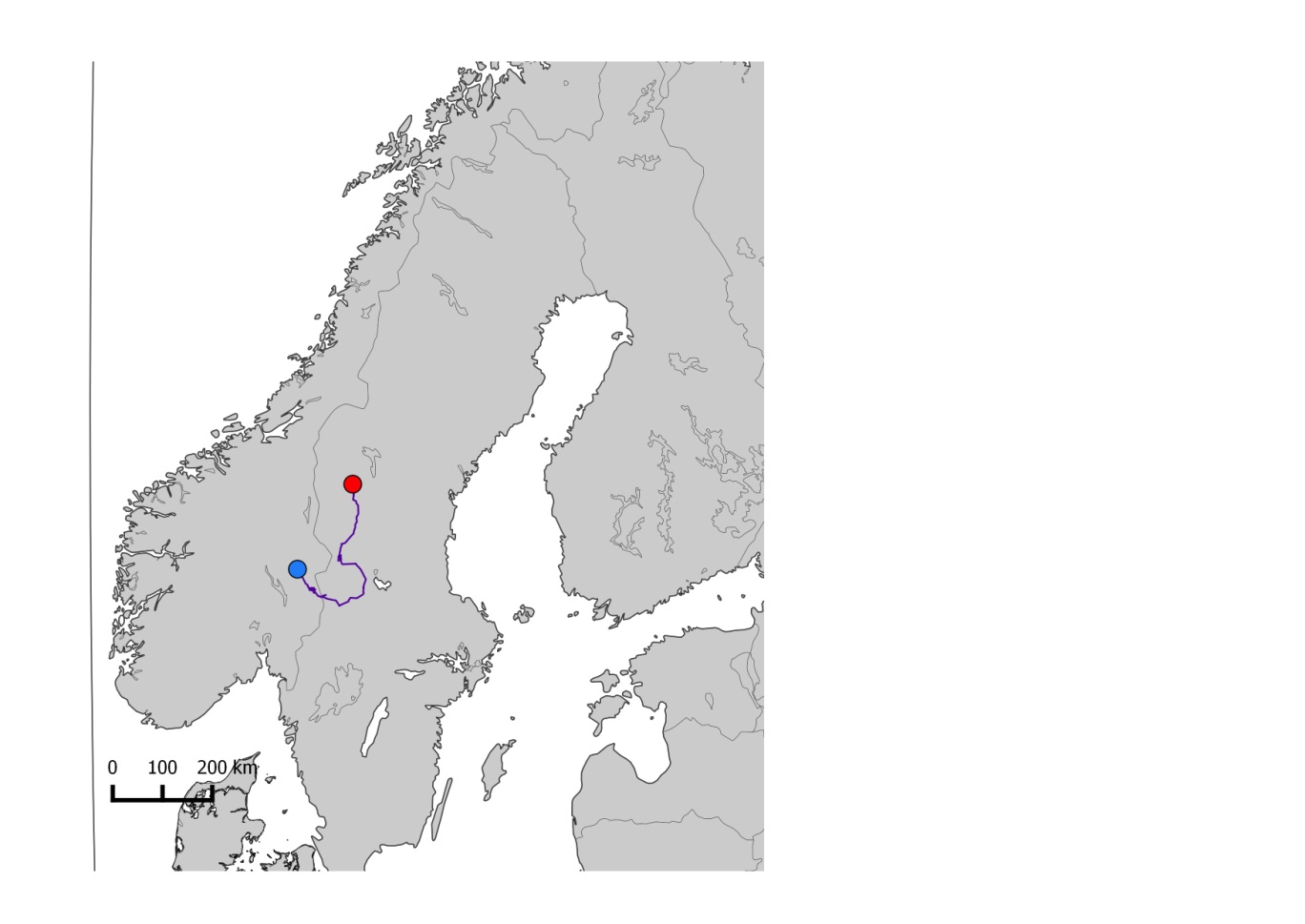


**Female M 09-08**

**Male M 14-06**

**Male M 09-12**

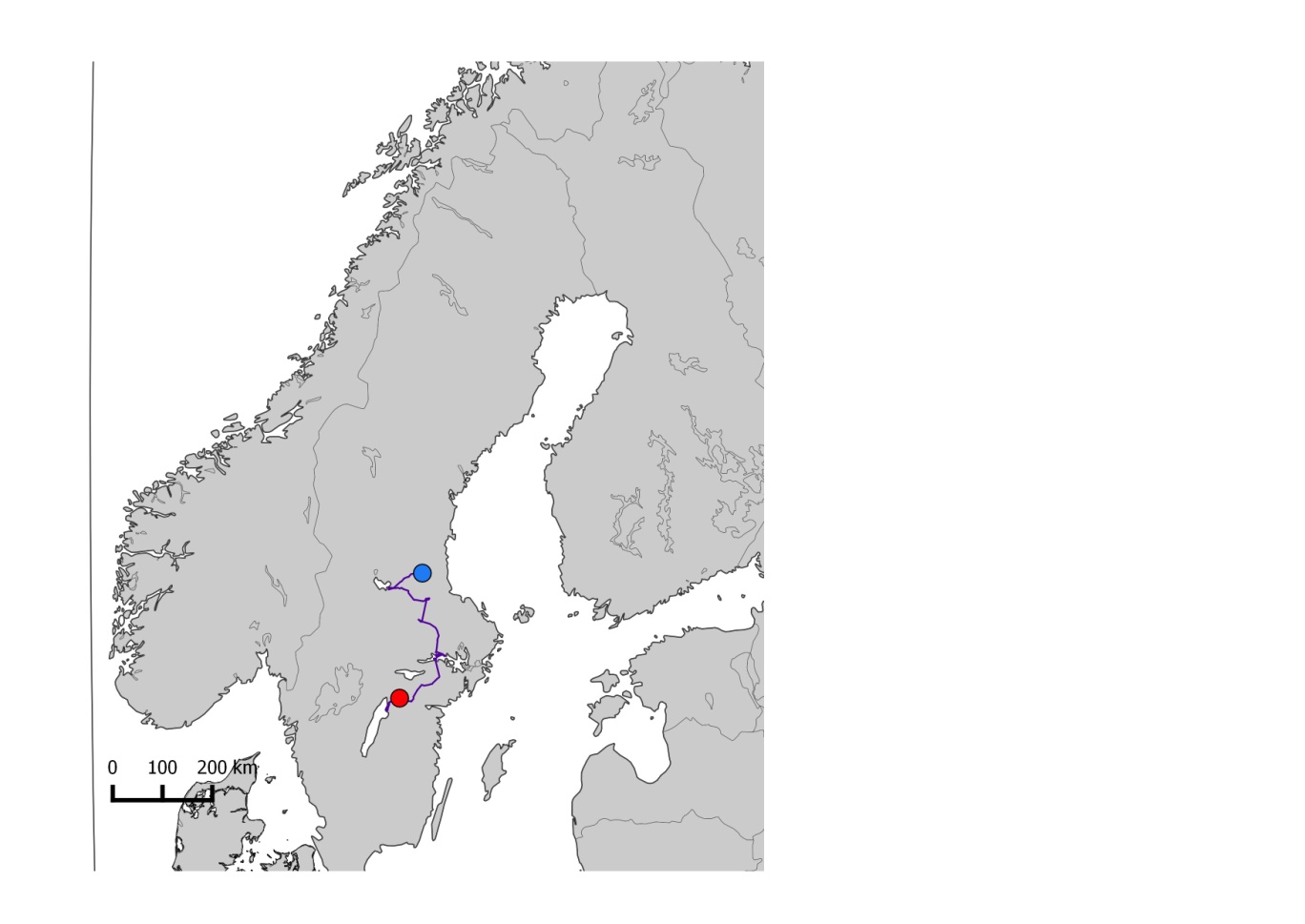


**Figure A2.** Real trajectories (maps) and dispersal movement characteristics (graphics) of the 11 medium and long-distance dispersing GPS-collared gray wolves in Scandinavia used to simulate trajectories from natal to newly established territories. The long trajectories were simulated from the individuals M 14-08, M 11-05, and M 03-01. The birth territory is represented in blue, and the established territory (for all individuals except for Female M 03-01, Male M 14-08, and Male M 09-12, for which we used positions from dispersal date to the date when the GPS-positioning system stopped functioning) is represented in red. The movement characteristics are speed (km/h) on the vertical histogram and turning angles (degrees) on the circular diagram.


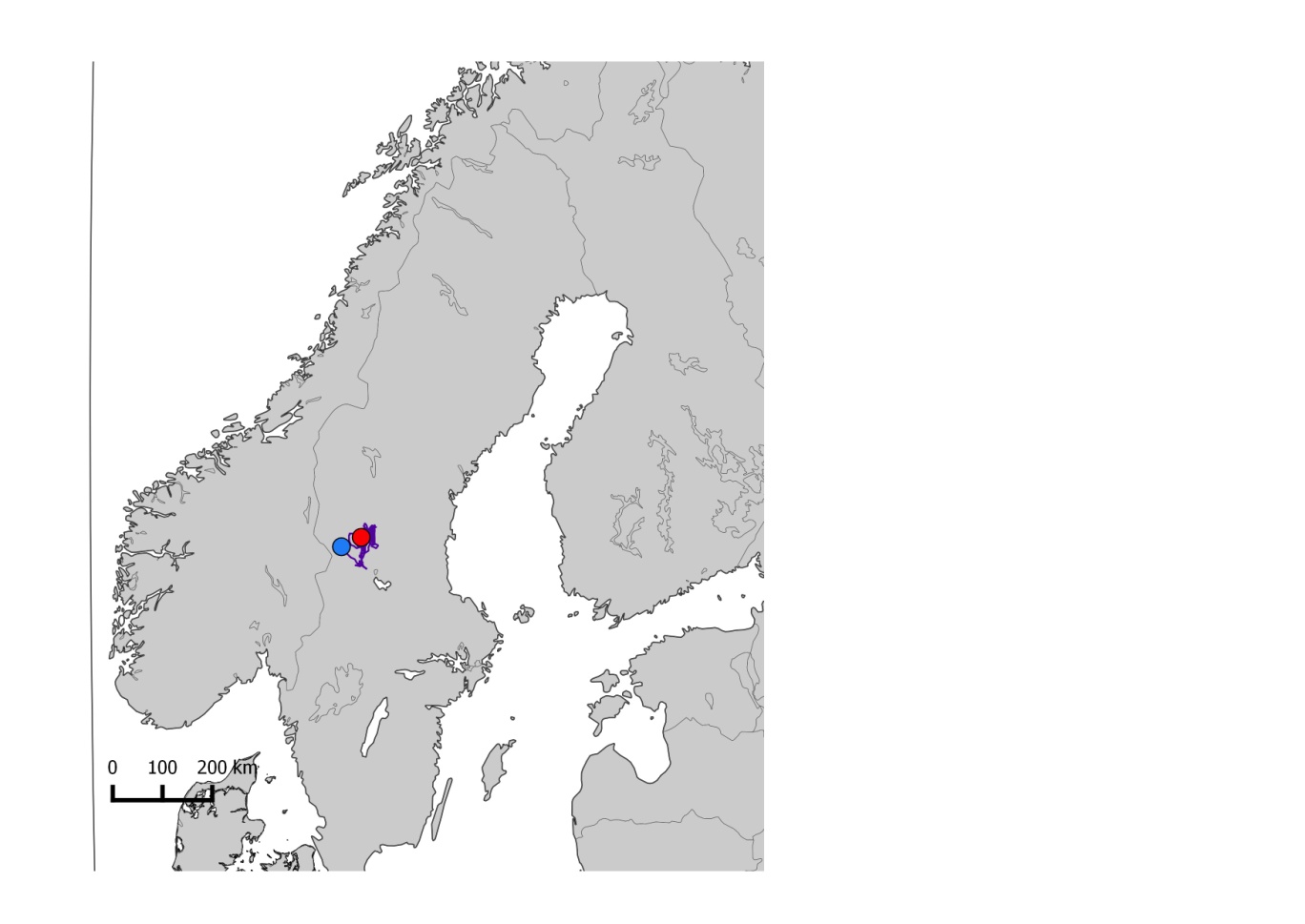

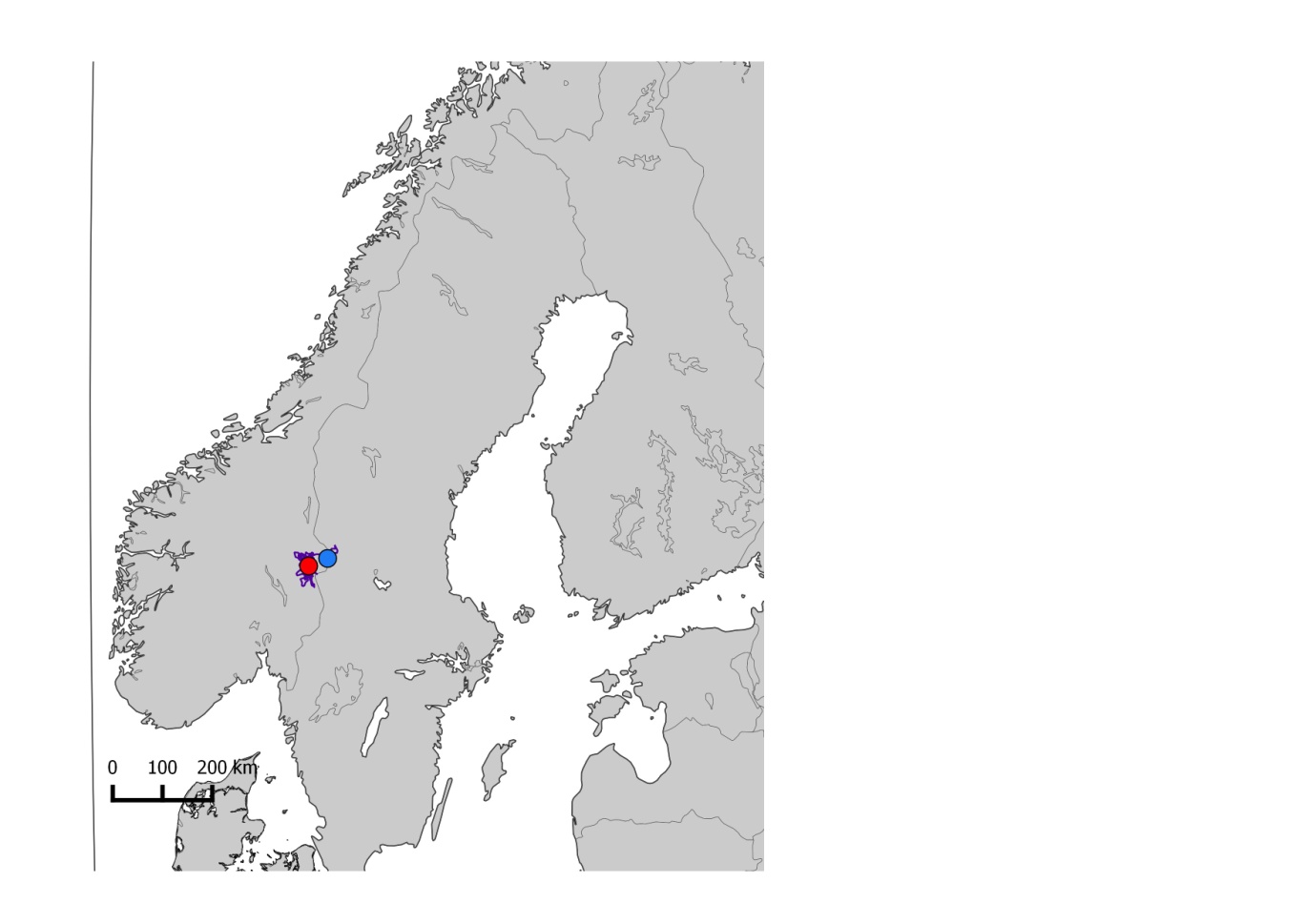


**Female M0909**

**Female M0907**

**Figure A3.** Real trajectories (maps) and dispersal movement (graphics) characteristics of short-distance dispersing GPS-collared gray wolves in Scandinavia used to simulate trajectories from natal to newly established territories. The birth and established territory are represented in blue and red, respectively. The movement characteristics are speed (km/h) on the vertical histogram and turning angles (degrees) on the circular diagram.

**
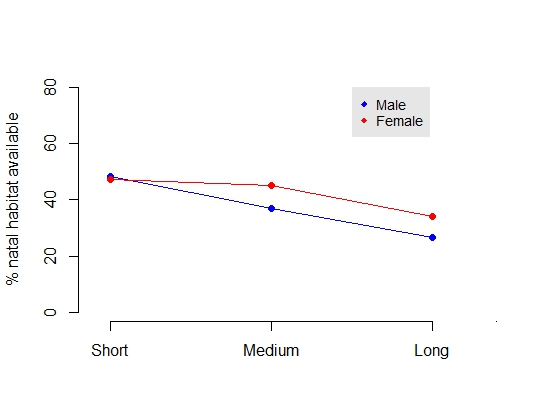
**

**Figure A4:** Average proportion of the random points defining availability that had similar habitat characteristics compared to the habitat of the natal territory for dispersing Scandinavian wolves. Availability was sampled using correlated random walks and habitat similarity defined using the k-means clustering approach with 6 clusters-division. The proportions are shown for each gender (red = female, blue = male) and for short (< 40 km), medium (40 - 200 km) and long (> 200 km) dispersers (x-axis).

**
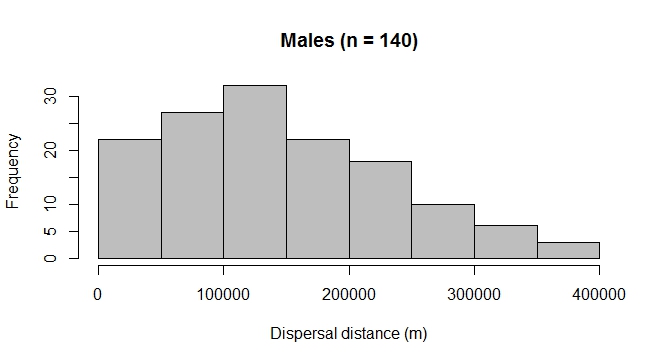

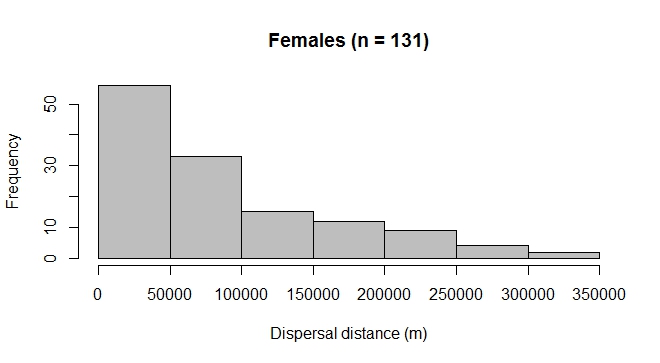
**

**Figure A5:** Distribution of the dispersal distances of Females (**A**) and Males (**B**) between the natal and established territories for the 271 studied wolves in central Scandinavia.

**
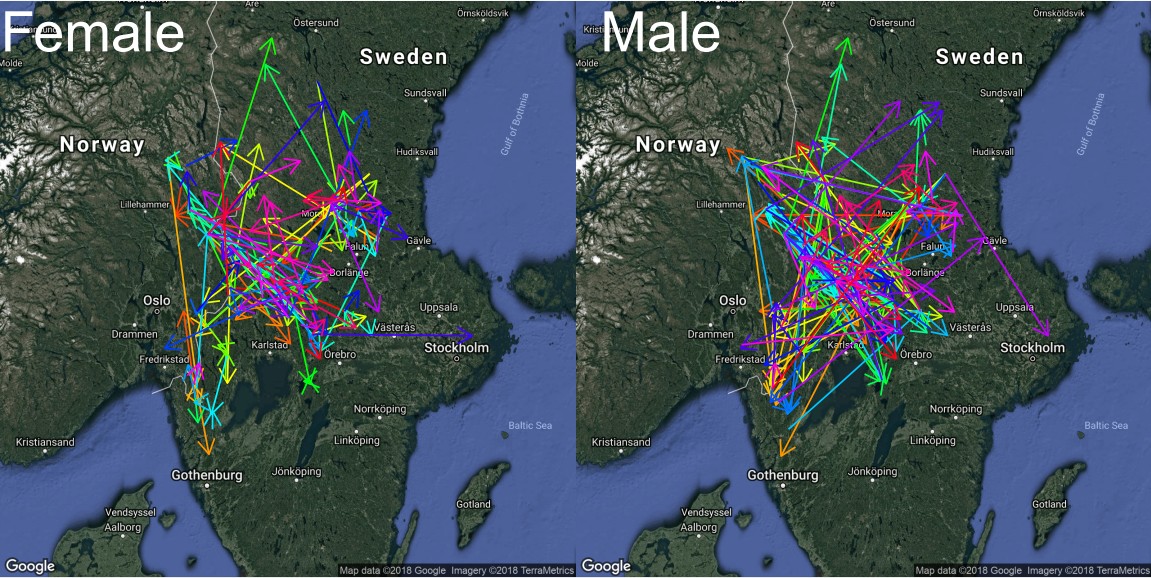
**

**Figure A6:** Dispersal maps of the studied Females (left Panel) and Males (right panel) wolves in central Scandinavia. The different colored arrows link the location of the centroid of a natal territory to the established territory for each individual.

**APPENDIX 3:**

**DEFINITION OF HABITAT SIMILARITY USING PCA AND K-MEANS CLUSTERING**

To determine whether wolves established in similar or different habitats than those characterizing the natal territory, we used the K-means clustering method over the five Principal Components of the PCA analysis, which explained 80% of the variance (Table A1). We used cluster validation measures to check for the best clustering method (R package clValid; 11), which recommended a K-means cluster analysis with a 6-cluster division (Fig. A7).

**Definition of cluster types**

Cluster 1 ("Rough terrain"), was characterized by terrain-related variables, i.e., mountain, altitude, mire, slope, and roughness (Fig. A7; Fig. A10, Appendix 5). In contrast, Cluster 2 ("Intermediate human") was characterized by intermediate levels of the human-related variables: human accessibility, human density, human dominated area, agriculture, and main and secondary roads. Cluster 3 ("High human") encompassed habitat types with high values of all the human-related variables. Cluster 4 ("Intermediate rough") was defined by intermediate levels of the terrain variables mountain, altitude, mire, slope, and roughness, with some influence of bear density. Cluster 5 ("Bear"), was mainly characterized by the variables bear density and mire. Cluster 6 ("Moose") was mainly characterized by the moose density variable, with some influence of slope, roughness, and main road. Fig. A8 shows the location of the clusters in the study area.

**Table A1**. Principal Component Analysis (PCA) on the matrix containing the environmental variables characterizing the natal, available and established territories of gray wolves in Scandinavia, 1998-2012. Variance and cumulative variance explained by the five components retained from the PCA based on the Kaiser method (12), i.e., the components with eigenvalues > 1.

|  | **Principal component number** | | | | |
| --- | --- | --- | --- | --- | --- |
|  | **1** | **2** | **3** | **4** | **5** |
| **Eigenvalue** | 5.99 | 2.31 | 2.28 | 1.11 | 1.04 |
| **Proportion of variance explained** | 37.45 | 14.45 | 14.24 | 6.95 | 6.47 |
| **Cumulative variance explained (%)** | 37.45 | 51.90 | 66.15 | 73.10 | 79.57 |

**Figure A7.** The principal component analysis (PCA) of natal, available, and established gray wolf territories in Scandinavia illustrated with the two first principal components **(A)** and the third and fourth components (**B)** and the proportion of variance explained (%). Variables are represented by arrows and the variable contribution to the PC is presented by the length of the arrow. All variables are summarized in Table 1 (main text). Six clusters of habitat types are represented by different colors and shapes. Each cluster contains natal, available, and established territories with similar habitat characteristics.

**
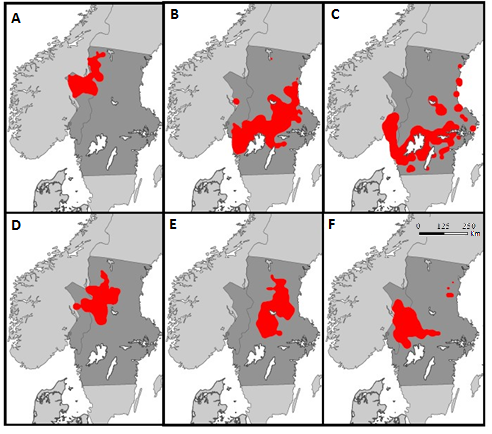
**

**Figure A8.** Spatial location of the clusters characterized by different habitat types in the Scandinavian wolf range, as identified by K-means clustering analysis. The red areas represent the location of wolf territories (natal, available and established) with similar habitat characteristics between 1998 and 2012. A 95% kernel contour was applied to locations of territories for each cluster: Cluster 1-"Rough terrain" (A), Cluster 2-"Intermediate human" (B), Cluster 3-"High human" (C), Cluster 4-"Intermediate rough" (D), Cluster 5-"Bear" (E) and Cluster 6-"Moose" (F).

**Table A2.** Average values (SD) of all landscape variables for each of the clusters describing the habitat types characterizing natal, established and available wolf territories in Scandinavia.

| **Variable** | **Rough terrain**  **(C1)** | **Intermediate human**  **(C2)** | **High**  **human**  **(C3)** | **Intermediate rough**  **(C4)** | **Bear**  **(C5)** | **Moose**  **(C6)** |
| --- | --- | --- | --- | --- | --- | --- |
| Human density | 1.84 (2.98) | 17.8 (11.42) | 64.53 (86.88) | 2.95 (3.29) | 7.22 (4.57) | 10.12 (6.49) |
| Human-dominated | 0 (0) | 0.02 (0.01) | 0.04 (0.02) | 0.01 (0.01) | 0.01 (0.01) | 0.01 (0) |
| Agriculture | 0.01 (0) | 0.1 (0.06) | 0.22 (0.11) | 0.01 (0.01) | 0.02 (0.02) | 0.06 (0.05) |
| Forest | 0.58 (0.13) | 0.74 (0.05) | 0.53 (0.11) | 0.74 (0.05) | 0.8 (0.04) | 0.8 (0.05) |
| Mire | 0.14 (0.05) | 0.03 (0.02) | 0.02 (0.02) | 0.19 (0.04) | 0.1 (0.04) | 0.06 (0.03) |
| Water | 0.03 (0.02) | 0.11 (0.04) | 0.18 (0.14) | 0.04 (0.02) | 0.07 (0.02) | 0.06 (0.03) |
| Mountain | 0.24 (0.13) | 0 (0.01) | 0.01 (0.02) | 0.02 (0.04) | 0 (0) | 0.01 (0.01) |
| Secondary road | 0.32 (0.12) | 0.98 (0.16) | 0.86 (0.26) | 0.61 (0.13) | 0.96 (0.09) | 0.84 (0.14) |
| Main road | 0.11 (0.03) | 0.36 (0.06) | 0.46 (0.13) | 0.2 (0.04) | 0.24 (0.04) | 0.32 (0.07) |
| Bear | 0.22 (0.17) | 0.1 (0.18) | 0.04 (0.13) | 0.43 (0.26) | 0.42 (0.3) | 0.06 (0.07) |
| Altitude | 738.5 (91.16) | 166.77 (57.01) | 92.69 (55.36) | 501.93 (81.03) | 308.82 (49.64) | 287.35 (78.45) |
| Slope | 4.91 (1.23) | 2.34 (0.63) | 1.51 (0.82) | 2.98 (0.65) | 2.61 (0.39) | 3.48 (0.45) |
| Roughness | 45.85 (11.77) | 23.14 (5.92) | 15.44 (8.15) | 28.2 (6) | 25.36 (3.76) | 33.25 (4.1) |
| Human accessibility | 18.02 (13.36) | 82.86 (45.07) | 131.9 (69.89) | 21.19 (17.02) | 34.06 (25) | 24.73 (15.98) |
| Moose | 0.2 (0.09) | 0.27 (0.1) | 0.25 (0.1) | 0.21 (0.09) | 0.19 (0.06) | 0.44 (0.07) |

**APPENDIX 4:**

**EFFECT OF HABITAT DEFINITION ON NHBD**

*Clustering*

To test if alternative definition of habitat similarity influenced our conclusion about NHBD, we conducted the same conditional logistic regression described in the methods section, but alternatively using different numbers of cluster-division and clustering methods. This was done using our two definitions of habitat availability, i.e., CRW (Fig. 2, main text) and increasing buffer sizes around dispersing individual wolves (Fig. 3, main text; Fig. A9). We used K-means with a number of clusters ranging from 4 - 10, which did not result in NHBD (Fig. 2, main text). We also used the same number of clusters as in the main analysis (6 clusters) by using Hierarchical Clustering methods (e.g., 13) with the "hclus" function and Partition Around Medoids (e.g., 14) with the "pamk" function from the "fpc" R package (15). Neither of these two clustering methods changed our results regarding NHBD (Fig. 2, main text).

*Distance metric*

We calculated a metric of similarity to the natal habitat. Based on the habitat matrix obtained for all observations (natal, established, and available territories), we calculated the Euclidean distance matrix by using the "dist" function from the "stats" R package (16). For each individual, we then extracted the distance metric from the natal territory to the established (1) and available (0) territories. We used this continuous metric (low value: similarity; high value: different) as the NHBD response variable in the logistic regression instead of the binary variable (0 Different; 1: Similar) used when habitat similarity was defined using the clustering approach. Using this metric of similarity in the conditional logistic regression did not change our results on NHBD either (Fig. 2, **
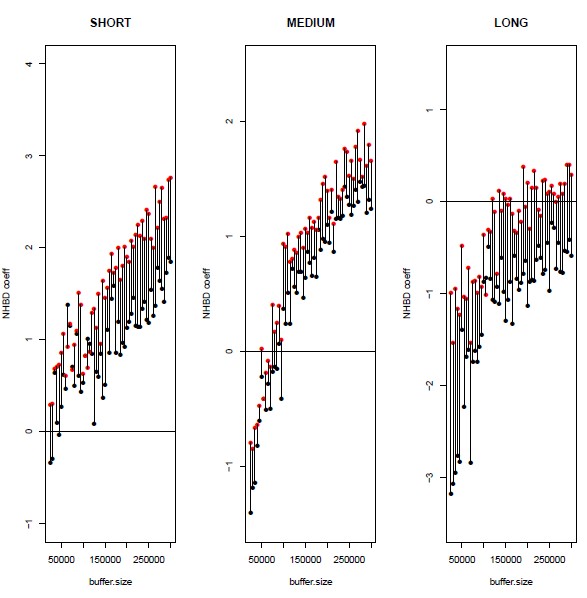
**
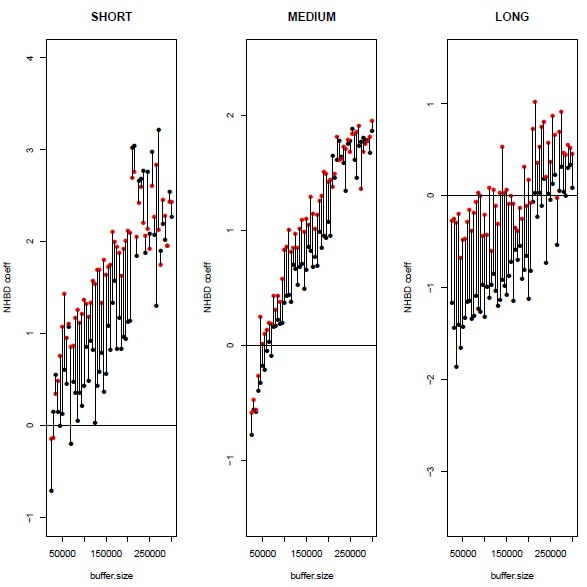
main text).

**b)**

**a)**


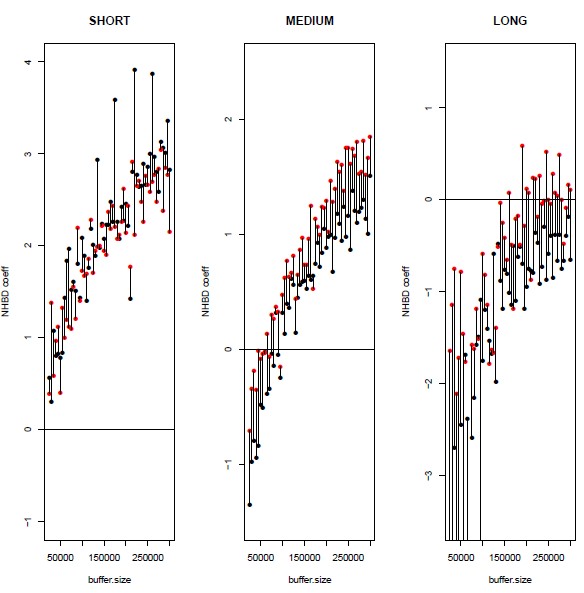

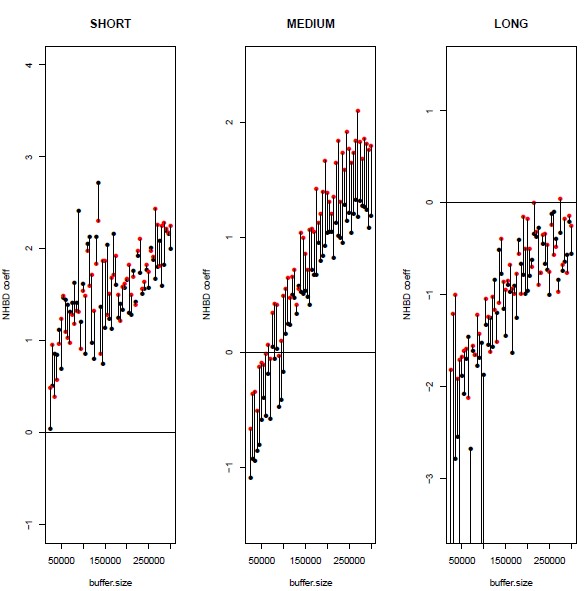

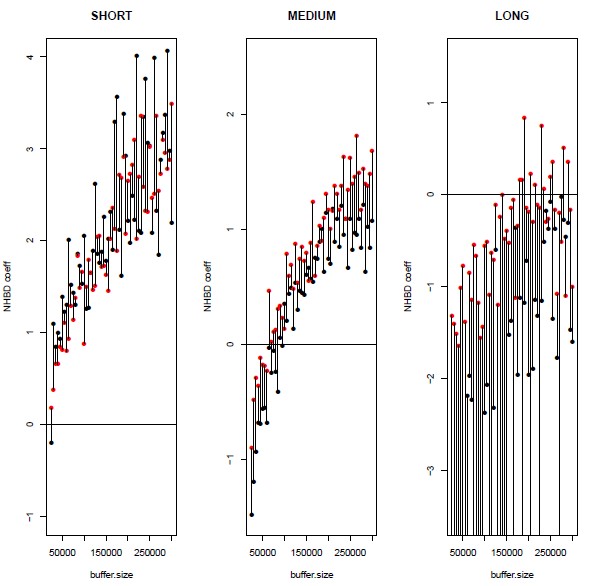

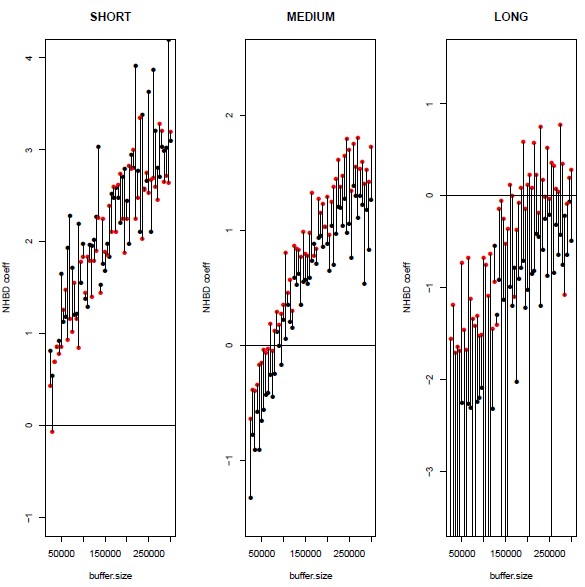

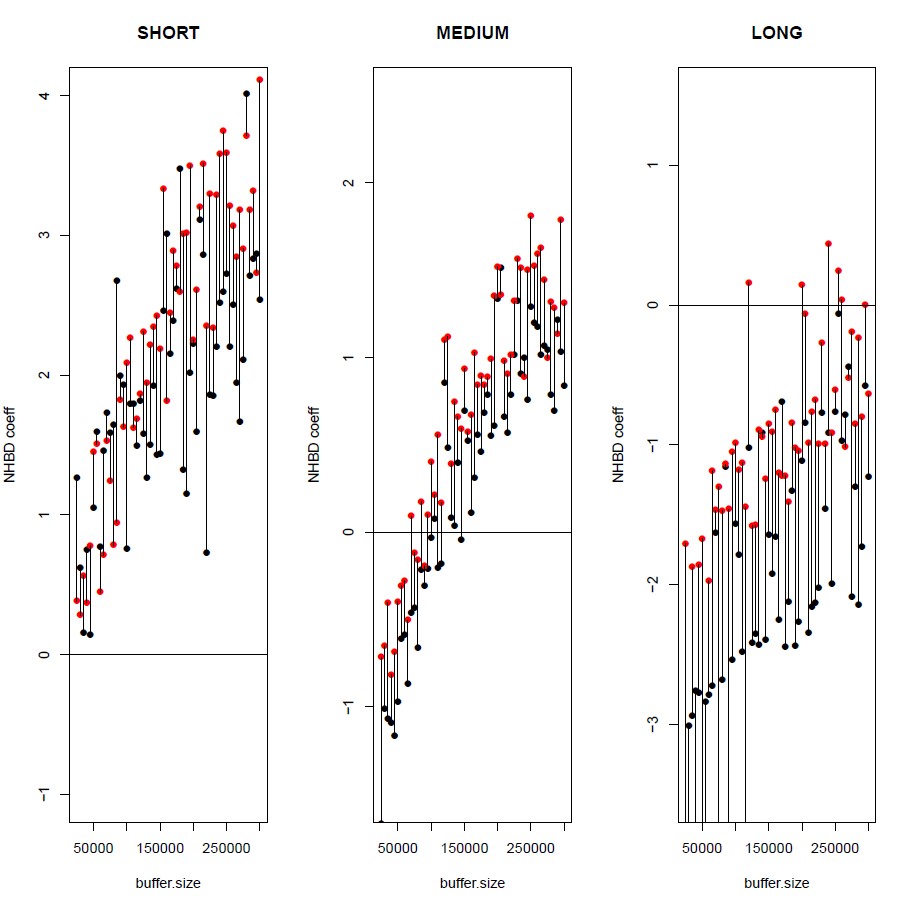

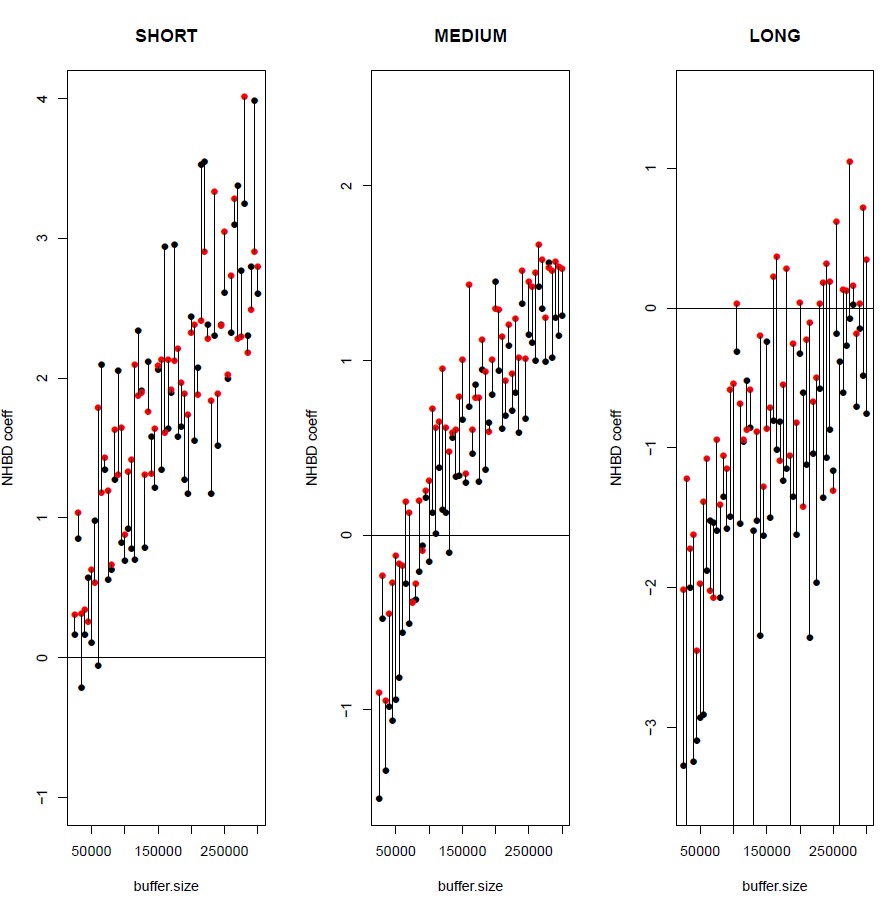


**d)**

**c)**

**e)**

**f)**

**h)**

**g)**


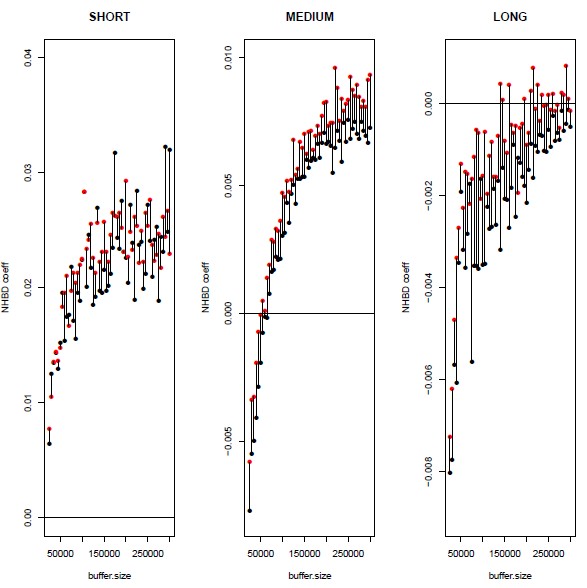


**i)**

**Figure A9.** Coefficients (β) of the probability of NHBD for male (black dots) and female (red dots) wolves in Scandinavia (1998 - 2012). Parameters were estimated from the conditional logistic regressions for short, medium and long dispersers. Availability was defined by using different buffer sizes. Figure panels represent the different methods of defining habitat similarity: a-f) 4, 5, 7-10 K-means cluster divisions (the 6 cluster division is shown in the main text); g) Hierarchical clustering methods; h) PAM; i) Distance metric. For the distance metric, the coefficients were inversed for purposes of readability and comparison with other methods. In its original scale, a negative β value of the NHBD variable using the metric distance is to be interpreted as individuals performing NHBD and positive β value as the opposite.

**APPENDIX 5:**

**SUPPLEMENTARY RESULTS**

We specifically addressed the role of environmental variables for the whole wolf population by performing the same analysis described in the Methods section, but without considering dispersal distances. We did this by defining habitat availability using CRW and habitat similarity using k-means with 6-cluster division (Fig. A7, A10). Sex was included as part of an interaction term with NHBD. We included the environmental variables used to define territory characteristics as predictors to control for avoidance/selection of specific habitat types. We tested for correlations and removed the variables human-dominated areas, agricultural lands, altitude, roughness, mires, and secondary roads from the model. We used this model in the Results and Discussion sections of the main text to interpret specific avoidance/selection patterns of individuals towards environmental variables.

## Natal territories from the whole studied wolf population were distributed along the 6 clusters obtained with the K-means clustering methods (Fig. A10). Wolves selected the natal habitat type in different proportions, depending on the natal cluster (Fig. A10). In this analysis, we did not find support for the NHBD hypothesis (β ± SE = -0.34 ± 0.31; p = 0.28; Table 2). Nevertheless, male wolves showed less NHBD than females (β ± SE = -0.80 ± 0.31; p < 0.01; Table 2). See main text for results on selection/avoidance oftowards specific environmental variables.

**Figure A10.** Spanning network summarizing the natal-habitat biased dispersal (NHBD)
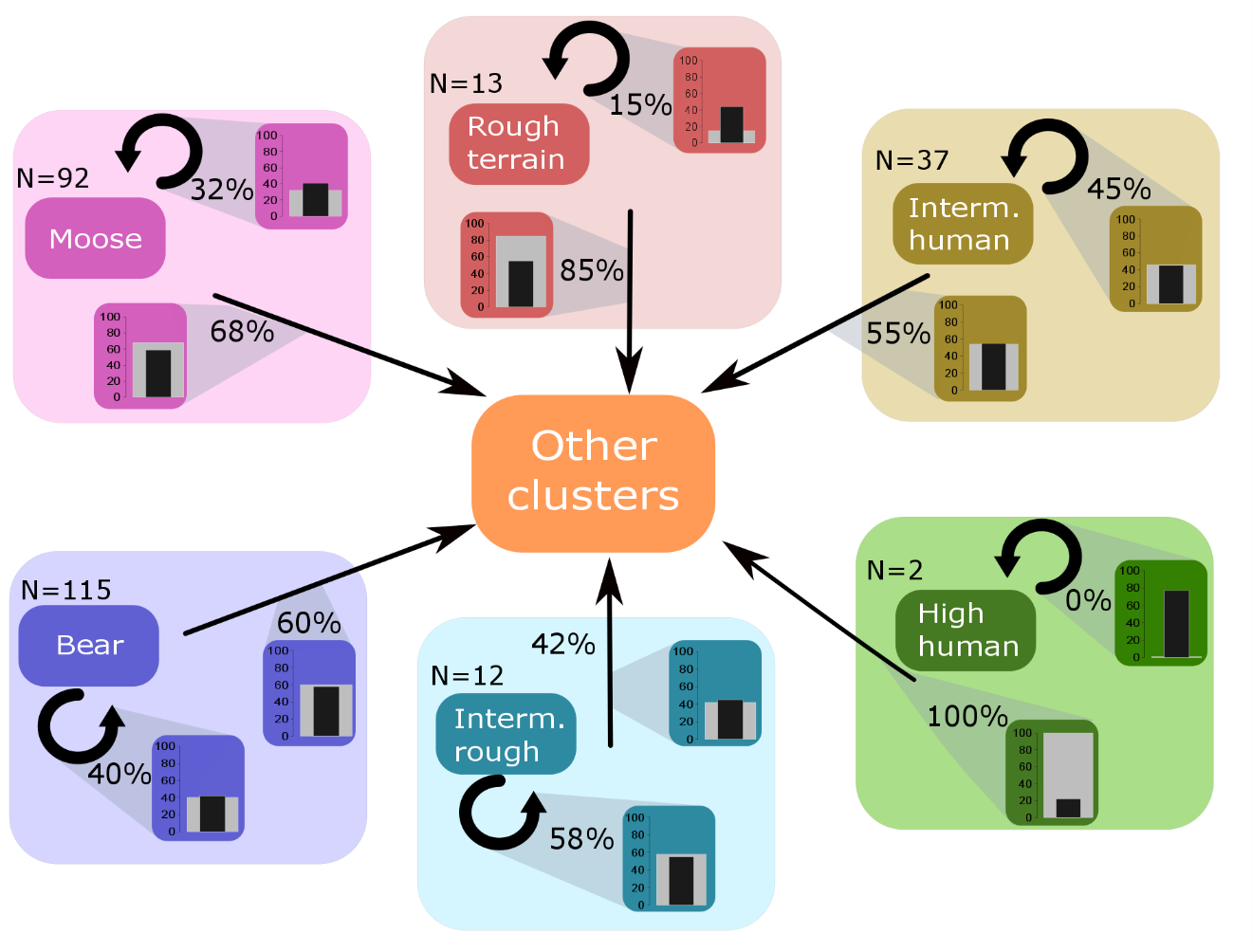
of gray wolves in Scandinavia, 1998 - 2012. Each of the six outer colored boxes represents the NHBD behavior (habitat types, i.e., *cluster* selected when establishing a territory) of wolves born in each of the six different clusters. The number of wolves born in each habitat type (N) is shown for each cluster. The circular arrow and its associated barplot represents the percentage of individuals that selected a territory of the same habitat type than the natal habitat (gray bar). The black barplot represents the availability of that type of habitat. The straight arrow and its associated barplot shows the percentage of individuals that selected a different habitat compared to the natal territory (gray bar). The black barplot represents the availability of other type of habitats. See Appendix 3 for a further description of the different types of clusters.

**REFERENCES**

1. Liberg O, Aronson Å, Sand H, Wabakken P, Maartmann E, Svensson L, et al. Monitoring of wolves in Scandinavia. 2012.

2. Liberg O, Andrén H, Pedersen H-C, Sand H, Sejberg D, Wabakken P, et al. Severe inbreeding depression in a wild wolf Canis lupus population. Biology letters. 2005;1(1):17-20.

3. Åkesson M, Liberg O, Sand H, Wabakken P, Bensch S, Flagstad Ø. Genetic rescue in a severely inbred wolf population. Molecular Ecology. 2016;25(19):4745-56.

4. Wabakken P, Sand H, Kojola I, Zimmermann B, Arnemo JM, Pedersen HC, et al. Multistage, Long‐Range Natal Dispersal by a Global Positioning System‐Collared Scandinavian Wolf. The Journal of wildlife management. 2007;71(5):1631-4.

5. Liberg O, Aronson Å, Sand H, Wabakken P, Maartmann E, Svensson L, et al. Monitoring of wolves in Scandinavia. Hystrix, the Italian Journal of Mammalogy. 2011;23(1):29-34.

6. Mattisson J, Sand H, Wabakken P, Gervasi V, Liberg O, Linnell JD, et al. Home range size variation in a recovering wolf population: evaluating the effect of environmental, demographic, and social factors. Oecologia. 2013;173(3):813-25.

7. Dettki H, Brode M, Giles T, Hallgren J. Wireless remote animal monitoring (WRAM)-A new international database e-infrastructure for management and sharing of telemetry sensor data from fish and wildlife. 2014.

8. Ordiz A, Milleret C, Kindberg J, Månsson J, Wabakken P, Swenson JE, et al. Wolves, people, and brown bears influence the expansion of the recolonizing wolf population in Scandinavia. Ecosphere. 2015;6(12):1-14.

9. Milleret C, Wabakken P, Liberg O, Åkesson M, Flagstad Ø, Andreassen HP, et al. Let's stay together? Intrinsic and extrinsic factors involved in pair bond dissolution in a recolonizing wolf population. Journal of Animal Ecology. 2017;86(1):43-54.

10. Milleret C, Wabakken P, Liberg O, Åkesson M, Flagstad Ø, Andreassen HP, et al. Let's stay together? Intrinsic and extrinsic factors involved in pair bond dissolution in a recolonizing wolf population. Journal of Animal Ecology. 2016.

11. Brock G, Pihur V, Datta S, Datta S. clValid: Validation of clustering results. R package version 06-4. 2011.

12. Coghlan A. A little book of R for multivariate analysis. Cambridge: Wellcome Trust Sanger Institute. 2013.

13. Castillo-Rodríguez M, López-Blanco J, Muñoz-Salinas E. A geomorphologic GIS-multivariate analysis approach to delineate environmental units, a case study of La Malinche volcano (central México). Applied Geography. 2010;30(4):629-38.

14. Long J, Nelson T, Wulder M. Regionalization of landscape pattern indices using multivariate cluster analysis. Environmental management. 2010;46(1):134-42.

15. Hennig C. fpc: Flexible procedures for clustering. R package version 2.1-5. 2013.

16. Team RC. R: A language and environment for statistical computing. R Foundation for Statistical Computing, Vienna, Austria. 2013. ISBN 3-900051-07-0; 2014.
